# Approximate Entropy in Canonical and Non-Canonical Fiction

**DOI:** 10.3390/e24020278

**Published:** 2022-02-15

**Authors:** Mahdi Mohseni, Christoph Redies, Volker Gast

**Affiliations:** 1 Department of English and American Studies, University of Jena, 07743 Jena, Germany; mahdi.mohseni@uni-jena.de (M.M.); volker.gast@uni-jena.de (V.G.); 2 Experimental Aesthetics Group, Institute of Anatomy I, Jena University Hospital, University of Jena, 07743 Jena, Germany

**Keywords:** Approximate Entropy, Shannon Entropy, fictional texts, non-fictional texts, canonical texts, non-canonical texts, POS-tags, text classification

## Abstract

Computational textual aesthetics aims at studying observable differences between aesthetic categories of text. We use Approximate Entropy to measure the (un)predictability in two aesthetic text categories, i.e., canonical fiction (‘classics’) and non-canonical fiction (with lower prestige). Approximate Entropy is determined for series derived from sentence-length values and the distribution of part-of-speech-tags in windows of texts. For comparison, we also include a sample of non-fictional texts. Moreover, we use Shannon Entropy to estimate degrees of (un)predictability due to frequency distributions in the entire text. Our results show that the Approximate Entropy values can better differentiate canonical from non-canonical texts compared with Shannon Entropy, which is not true for the classification of fictional vs. expository prose. Canonical and non-canonical texts thus differ in sequential structure, while inter-genre differences are a matter of the overall distribution of local frequencies. We conclude that canonical fictional texts exhibit a higher degree of (sequential) unpredictability compared with non-canonical texts, corresponding to the popular assumption that they are more ‘demanding’ and ‘richer’. In using Approximate Entropy, we propose a new method for text classification in the context of computational textual aesthetics.

## 1. Introduction

Computational textual aesthetics is an emerging field at the interface of literary studies and linguistics. This field aims at identifying the statistical properties of texts to reflect categorizations of different types, e.g., authorship [1,2] and genre [3,4]. From the perspective of empirical aesthetics, properties that can potentially be associated with aesthetic categories and with perceptual responses during reading are of particular interest, as they can provide a basis for formulating specific hypotheses for experimental studies. The present study was inspired by research in (experimental) visual aesthetics, a well-established field with a tradition reaching back to the 19th century [5,6].

More recently, several computational algorithms have been proposed for the analysis of statistical properties in visually pleasing images, including visual artworks, in comparison to images with less aesthetic appeal. Particular emphasis in the studies on artworks has been on global image properties that reflect artistic composition [7]. Many of these properties reflect various aspects of fractality/self-similarity, predictability and variability in the distribution of pictorial elements across individual images. Such properties are believed to form a perceptual basis of aesthetic responses and, hence, of judgments concerning the aesthetic value of an image [8].

The question arises whether texts, like images, are characterized by global properties correlating with the aesthetic responses to those texts during reading. This question is motivated by the hypothesis of an analogy between visual processing and reading [9] on the basis of the assumption of domain-general perceptual and cognitive components in linguistic processing [10]. Studying the aesthetic responses to texts directly would require comprehensive investigations including the observation of reader behaviour during reading (see for instance [11]). As a first step towards this program, we study the structural properties of texts grouped into different aesthetic categories. Such studies can form the basis of experimental investigations at a later stage and provide important cues concerning the experimental design, e.g., with respect to the stimulus material used and the variables analysed.

Previous observational research in textual aesthetics has often focused on poetry. While most of this research is exploratory and there is still work to be done, a number of interesting observations have been made. For example, Simonton [12] compared the vocabulary of the more “obscure” and the more popular sonnets of Shakespeare. He found a correlation between the lexical diversity and the “aesthetic success” of the sonnets. Forsyth [13] analysed the lexical features, vocabulary richness and the frequency distribution of syntactic tags in poems. He showed that the more popular poems generally used shorter words, fewer rare words, more coordinating conjunctions and more personal pronouns. Kao and Jurafsky [14] studied the style and content of poems written by professional and amateur poets to identify textual features associated with poetic beauty. Their analysis showed that more prestigious poets tended to refer more frequently to natural objects. Moreover, they made less reference to abstract concepts and used more ordinary and common words, though their vocabulary was richer.

The aesthetics of prose texts has been studied by relying on data from websites or social networks. Ashok et al. [15] attributed the success of novels to the writing style. They operationalized ‘success’ as the number of downloads from the Project Gutenberg site, using the distribution of POS-tags, grammatical rules, constituents and sentiments as basic measurements. In this way, they managed to classify more successful and less successful novels of different genres with acceptable accuracy.

Maharjan et al. [16] operationalized the success of a novel in terms of the average ratings on Goodreads, a social network for book lovers. They used “hand-crafted” textual features, such as the lexical and syntagmatic properties, sentiments and readability measures to predict the success of novels. Maharjan et al. [17] approached the classification of (un)successful novels by modelling the flow of emotion along a book. They showed that emotional information predicted the success of a text with relatively high accuracy.

While entropy measures have mostly been used to analyse the distributional laws of linguistics, e.g., concerning word order [18,19,20,21] and word length [22,23,24,25], or for a comparison of languages in terms of ordering preferences and complexity [26,27,28,29,30], there are also studies that investigated the aesthetic preference and popularity of texts using entropy metrics.

Febres and Jaffe [31] analysed the entropy and symbolic diversity of literary texts written by English and Spanish Nobel laureates and non-Nobel laureates. Their analyses confirmed that there was a correlation between the global statistical properties of texts and the two categories of authors. Chang et al. [32] analysed Shakespeare’s and Jin Yong’s works using a metric called “information-based energy”. They showed that the more popular works had a higher “energy”.

One of the main challenges of textual aesthetics is the question of how we can capture the global properties of longer prose texts, such as novels. Previous studies have used Multifractal Detrended Fluctuation Analysis as a way of measuring fractality or long-range correlations in texts. Drożdż et al. [33] analysed the fractality of sentence-length series in a corpus of Western fictional texts. Mohseni et al. [9] used a number of textual properties (sentence length, frequencies of specific POS-tags per sentence, lexical diversity measured with MTLD and topic probabilities) to generate series. They analysed these series in terms of variance and long-range correlations.

The numerical results of these methods were used as features in a classification task, intending to distinguish fiction from non-fiction and, within the fictional category, canonical vs. non-canonical English texts. The accuracy of classification was relatively high. This finding demonstrates the feasibility and usefulness of analysing the global structural design patterns of text. Of particular interest in this context are features that are amenable to experimental studies, specifically if they allow for an interpretation in terms of perception and processing, as has been hypothesized for fractality and long-range correlations [9].

Another important aspect of aesthetic perception is the degree of (ir)regularity in a text and, related to this, the degree of predictability or surprise in the signal—cf. Zipf’s principles of ‘unification’ and ’diversification’. Zipf [34] distinguished between the two antagonistic forces of ‘unification’ of the vocabulary, an economy principle from the speaker’s point of view of minimizing the number of word types used, and ‘diversification’, maximizing the fit between words and meanings and thus benefiting the listener (see also [35]). While unification and diversification in this sense are clearly related to predictability and surprise, at least from the point of view of the specific words used (but not the meanings), we assume that literary writing is not primarily driven by the principle of unification from the author’s point of view.

From an aesthetic point of view, a high degree of regularity/predictability is likely to facilitate processing, with a potentially positive effect on aesthetic perceptions. However, too much regularity may cause an impression of monotonicity. We therefore expect prose texts to reflect a trade-off between predictability and surprise. Moreover, we expect different text categories to assign different weights to two antagonistic design principles: “Keep it simple” and “Avoid monotonicity”. In other words, we expect different types of balance between predictability and uncertainty in canonical and non-canonical texts. Trade-offs of this type have also been observed in music perception [36,37].

In the present study, we are primarily concerned with fictional prose. The main objective is to identify the global structural properties of texts that we have classified into the categories of ‘canonical’ vs. ‘non-canonical’. This categorization is intended as an operationalization of aesthetic preference at a community level. While there is clearly a considerable degree of variation in individual taste, canonization—a process that involves a range of stakeholders from various sectors of society, such as literary scholars and publishers—reflects the taste of an ‘average educated reader’, and it has high prestige [38,39,40].

Canonical texts were written by skilled, mostly professional writers targeting an educated audience. Canonical literature is read in school, and educated members of societies are expected to be familiar with the major canonical works of their culture. In some countries, literary canons play an important role in the constitution of national identity (e.g., ‘national poets’, such as William Shakespeare (‘The Bard’) in the UK, Goethe and Schiller in Germany, Pushkin in Russia, etc.). Non-canonical texts do not have any of the prestige characteristics of canonical texts.

The central question of this study is whether, or to what extent, canonicity as a social attribute has structural correlates in the relevant texts. We focus on predictability and surprise, for the reasons mentioned above. As reading is a learned skill, we expect canonical texts to lean in the direction of surprise (“Avoid monotonicity”), at the expense of ease of processing. Non-canonical texts, by contrast, are (supposedly) written by less skilled writers, and not necessarily for a ‘trained’ audience. In this case, we expect ease of processing to prevail (“Keep it simple”). Note that we do not expect the relationship between predictability/surprise and the text categories ‘canonical’ and ‘non-canonical’ to be consistent. Our hypothesis is more general, in the sense that we expect the two classes to be associated with different balances between the two design principles of “Keep it simple” and “Avoid monotonicity”, potentially in different aspects of structural design.

The canonical and non-canonical texts of our corpus belong to the same genre, i.e., fictional prose. In order to gauge the degree of register specificity of the observed patterns, we included texts from a different register as well, i.e., non-fictional (expository) prose (see Section 2.1). We used two types of observables, the length of sentences and frequency distributions of part-of-speech (POS) tags. The frequencies of POS-tags were determined in fixed-size windows of text, which we call ‘boxes’ (see Section 2.2). To measure the (ir)regularity and predictability in a text, we used two types of entropy measures, Approximate Entropy (ApEn) and Shannon Entropy (ShEn) (see Section 2.3). Section 3 presents the results, which are then discussed in Section 4.

## 2. Data and Methods

### 2.1. The JEFP Corpus 2.0

For our computational textual aesthetics studies, we needed a corpus that was tailor-made for the purpose of the project, the comparison of canonical and non-canonical fiction. While there are several corpora of literary texts available (e.g., the Standardized Project Gutenberg Corpus/SPGC [41]), we compiled a corpus of our own with a certain balance across text types and the time of publication: the *Jena Expository and Fictional Prose* (JEFP) corpus. This corpus contains canonical and non-canonical fictional as well as non-fictional texts.

In a previous study [9], we used version 1.0 of this corpus. For the present study, we extended the corpus and included more texts, primarily in order to achieve a better balance in terms of the years of publication.

The canonical texts of the JEFP corpus 2.0 are the same as those contained in version 1.0. The corpus comprises 76 canonical literary texts from 30 authors, which were taken from the *Corpus of the Canon of Western Literature* (CCWL) [42], which, in turn, relies on Bloom [43] (*The Western Canon: The Books and School of the Ages*). As an additional criterion of canonicity (of authors), we used evidence from Wikipedia sites. We determined the number of articles for authors in the top 30 language editions of Wikipedia, as an approximate indication of their international reputation.

In order to obtain a sample of non-canonical fictional texts, we used the websites www.goodreads.com (accessed on 8 February 2022), feedbooks.com as well as Project Gutenberg (www.gutenberg.org, accessed on 8 February 2022). The raw texts were all extracted from the Project Gutenberg site.  We selected only long books that comprised at least 35,000 words, as a critical number of words is required for analysis of the global properties of text using methods such as Multifractal Detrended Fluctuation Analysis (MFDFA; see [9]).

At the time of compilation of the corpus (May 2020), none of the books classified as “non-canonical” by us had a download number higher than 40. By thresholding the download count, we avoided including non-canonical popular literature. In previous studies, download counts at the Project Gutenberg site have been used as a surrogate to gauge the success of books [15,44].

We made sure that the relevant (non-canonical) authors were not listed in the canon underlying our study, the Canon of Western Literature [43]. Moreover, none of the non-canonical authors has as many Wikipedia pages as the canonical author with the lowest number of pages (14). The authors classified as ‘non-canonical’ thus did not have the international prestige that is characteristic of canonical authors. The sample of non-canonical texts thus compiled contained 130 texts.

Non-fictional texts were also taken from the Project Gutenberg site. The sample contained in version 1.0 of the corpus was extended with texts from different genres, such as architecture, astronomy, geology, geography, philosophy, psychology and sociology. The extended corpus contained 185 texts of this category. Table 1 provides summary statistics for the texts of the corpus. The texts with metadata are listed in Supplementary Table S1.

For preprocessing of the texts, we removed the tables of contents and indices as well as any other material not belonging to the core text from each document. We cleaned up the texts semi-automatically using regular expressions, e.g., in order to rejoin hyphenated words and fix broken lines. We used the Stanza package for Python [45], an up-to-date neural-based text processing toolbox, to sentencize, tokenize and POS-tag all texts.

The three text categories of the JEFP corpus allowed us to carry out intra-genre comparison, i.e., canonical vs. non-canonical fictional texts, which is the main focus of our study, as well as inter-genre comparison, i.e., fictional vs. non-fictional texts. The inter-genre comparison is intended to give us an idea of the degree of genre specificity of any observed effects (see Section 3).

### 2.2. Properties Underlying Textual Structure

As reflexes of the structural organization of the texts, we used the length of sentences and part-of-speech tags (POS-tags) as assigned by the Stanza package for Python [45]. The distributions of POS-tags reflect grammatical structure as well as register and discourse modes [46]. For example, pronouns are associated with interactive communication, such as face-to-face conversation, verbs are typical of narration, and adjectives are characteristic of description. Regularity or irregularity in the organization of discourse modes can thus be measured in terms of the sequential distribution of POS-tags in a text.

In our study, we focused on six major parts of speech: nouns, verbs, adjectives, adverbs, pronouns and prepositions. We only took the top-level categories into account. For example, the tag ‘Noun’ covers singular as well as plural nouns and common nouns as well as proper names; different forms of verbs, such as the base forms, past tense forms and gerunds, are treated as a single class, ‘Verb’; simple, comparative and superlative adjectives are all subsumed under ‘Adjective’; and so on. We capitalize these general POS-tags in order to distinguish them from elements of the relevant classes (nouns, verbs, etc.).

We determined the frequencies of POS-tags per fixed-length segments, i.e., windows, of text. We did not use sentences as the scope of measurement because sentence length figured as a separate explanatory variable in our study and because we wanted to obtain measurements that were independent of punctuation practice. We therefore split the texts into windows of 25 tokens, which is the approximate average sentence length of the corpus (in fact, 23.3 tokens). It is important to mention, however, that the window size, within reasonable limits, did not have a noticeable effect on the results. We experimented with segments of 10 to 50 tokens in steps of 5 tokens but did not observe any major differences.

By windowing, each text is converted into a sequence of small bags of words—‘boxes’, as we call them—in which POS-tag frequencies are determined regardless of the position of the individual words. The linear order of the values obtained from the 25-words boxes was important as it was regarded as a reflex of the structural organization of the texts.

If the linear order of the counts is taken into account, as in the case of Approximate Entropy, we will speak of a ‘sequence of boxes’; if linear order does not matter, as in the case of Shannon Entropy, we will speak of a ‘bag of boxes’. Our approach is thus neither a bag-of-words nor a word-sequence approach. Word-sequence approaches—specifically, function–word-adjacency networks (WANs)—have been used in authorship attribution [47,48] and gender classification [48] (for a detailed description of WAN, see [49]).

As we used six parts of speech, we obtained six series based on counts of POS-tags in boxes. A series $X_{POS}=x\left(1\right),x\left(2\right),\cdots ,x\left(n\right)$ for a specific POS-tag thus contains the frequencies of the relevant tag in subsequent windows of 25 tokens. If *L* is the text length, the length of the series n=⌊L/25⌋. In the same way, we generated series of integers representing the length of the sentences in a text. Sentence length was measured as the number of tokens (including punctuation marks) in a sentence as sentencized by the Stanza package.

### 2.3. Computation of Unpredictability in Text

Each series generated as described in Section 2.2 is a sequence of events that are not independent from each other. As was shown in Mohseni et al. [9], they exhibit long-range correlations (though the method used to generate the series was slightly different in this publication). As an operationalization of (ir)regularity and predictability in a text, we used Approximate Entropy (ApEn), which measures predictability in linearly ordered random variables. We analysed sequences of POS-tag counts observed in ‘boxes’ in terms of Approximate Entropy (the ‘sequence-of-bags approach’).

In order to determine to what extent observed degrees of (ir)regularity are a property of the global (bag-of-boxes) distribution of structural features, rather than their linear arrangement, we also calculated summary statistics by using standard Shannon Entropy (ShEn). Associations of entropy values (ShEn and ApEn) with text categories were determined with a classification task, using a Support Vector Machine (Section 3.2). In what follows, we briefly describe both entropy measures, starting with Shannon Entropy.

#### 2.3.1. Shannon Entropy

Shannon Entropy (ShEn) is a well-known concept in information theory that measures uncertainty in a random variable. Given a discrete random variable *x* and a probability distribution p(x), the ShEn of *x*, h(x), is computed as

(1) $$h\left(x\right)=-\sum_{x\in S_{x}}p\left(x\right)\log_{e}p\left(x\right)$$

where $S_{x}$ is the set of all possible events. In a system with all possible events being equally likely to happen, uncertainty and, hence, ShEn, is at a maximum. A major advantage of ShEn is that it is parameter-free, straightforward and easily interpretable.

We can determine the ShEn for the six POS-series as well as the series of sentence length measurements and treat them as a global measurements of predictability. Once again, it should be stressed, however, that ShEn does not capture local patterns of distribution but is a function of the probability distribution as a whole. We therefore use it in conjunction with the Approximate Entropy as described in Section 2.3.2.

#### 2.3.2. Approximate Entropy

Approximate Entropy (ApEn) was first proposed by Pincus [50] as a way of measuring the degrees of regularity in times series. A high value of ApEn means a low degree of predictability and vice versa.

ApEn is computed according to sub-sequence matches of length *m* compared with sub-sequence matches of length m+1. The match between sub-sequences of a series is a function of a distance metric in relation to a predefined threshold value *r*. Let X=x(1),⋯,x(n) be a time series, *m* be the length of a sub-sequence and *r* be a positive value. ApEn is computed as follows:

1. Create sub-sequences $y_{i}^{m}=\left[x\left(i\right),\cdots ,x\left(i+\left(m-1\right)\right)\right]$ for i=1,⋯,n−m+1.
2. Using the distance between $y_{i}^{m}$ and $y_{j}^{m}$, defined as $d_{i,j}^{m}=\max_{k}\left|y_{i}^{m}\left(k\right)-y_{j}^{m}\left(k\right)\right|$, compute
  $$C_{i}^{m}\left(r\right)=\frac{1}{n-m+1}\sum_{j=1}^{n-m+1}𝟙\left(r-d_{i,j}^{m}\right)$$
  in which 𝟙(.) is the Heaviside function whose value is 1 when its parameter is positive and otherwise 0.
3. Compute
  $$\phi^{m}\left(r\right)=\frac{1}{n-m+1}\sum_{i=1}^{n-m+1}\log\left(C_{i}^{m}\left(r\right)\right)$$
4. Finally, calculate ApEn as
  $$\mathrm{ApEn}\left(m,r\right)=\phi^{m}\left(r\right)-\phi^{m+1}\left(r\right)$$

If the series is fixed at some value and is thus fully predictable, ApEn is 0. The value of ApEn depends on its two parameters, *m* and *r*. *m* is usually set to 2, and the value of *r*, which should be related to the standard deviation (SD) of the series, is set to 0.2 × SD (see, for example, [51,52,53]). In our experiments, we also applied this parameter setting.

ApEn has been subject to a broad range of research, and its behaviour has been studied under various types of circumstances. Researchers have proposed extensions of ApEn, such as Sample Entropy [54], Multi-Scale Entropy [55] and Multivariate Multi-Scale Entropy [56], which may provide more accurate analyses for certain time series. In order to compare ApEn with these extensions, we conducted experiments using the NeuroKit2 python package [57], which implements these extensions. We observed that none of these methods provided a better discrimination power compared to ApEn. Therefore, we only report the experimental results of ApEn in Section 3.

## 3. Results

In this section, we first present the results of the statistical analyses (Section 3.1) and then turn to the results of our classification experiment (Section 3.2). As pointed out in Section 2.1, the JEFP corpus contains texts from three categories: fiction/canonical, fiction/non-canonical, and non-fiction. Our main focus is on the difference between canonical and non-canonical fiction. As we wish to determine to what extent any observed differences are genre-related, we also included non-fictional texts in our comparison.

### 3.1. Statistical Analysis of Features

For each text in the corpus and for each text property, ApEn (Table 2) and ShEn (Table 3) were computed. As some features were not normally distributed (confirmed by a Kolmogorov–Smirnov test), we used the median values and compared them with the Mann–Whitney U test. In Table 2 and Table 3, each pair of columns shows a comparison of ApEn and ShEn values for each text category/feature combination. Whenever a value is significantly higher than the corresponding value for the other text category, the higher value is shown in bold face. Levels of significance are indicated by the superscripts on the right value within each pair of columns.

The most important observation that stands out from a superficial inspection of Table 2 and Table 3 is that the left two columns, which show the values for canonical and non-canonical fiction, exhibit a rather uniform pattern: while there are no significant differences between the values for sentence length (in the top row), the ApEn as well as the ShEn values for all series derived from POS-frequencies within boxes are higher for canonical than for non-canonical texts.

In contrast, in the right pair of columns, showing the comparison between fictional and non-fictional texts, there is no uniform pattern. Fictional texts have higher ApEn and ShEn values than non-fictional texts for Adverb and Pronoun, and the ApEn value for Noun is higher in fictional than in non-fictional texts. Non-fictional texts have higher ApEn and ShEn values for Verb, Adjective and Preposition, and the ShEn value for Sentence Length is higher than for fictional texts.

In conclusion, Table 2 and Table 3 thus show that entropy values—both ApEn and ShEn—are consistently higher in canonical than in non-canonical fiction for POS-tag frequencies within boxes, whereas there is no such clear tendency in the comparison between fictional and non-fictional prose (though there are also significant differences).

Stated differently, the results shown in Table 2 and Table 3 suggest that canonical fictional texts are characterized by a higher degree of uncertainty than non-fictional texts, when treated either as a bag-of-boxes distribution (with ShEn) or a sequence-of-boxes distribution (ApEn). Fictional texts differ from non-fictional texts in terms of the uncertainty associated with specific POS-tags; however, there is no uniform pattern. It appears that, in fictional prose, pronouns and adverbs are distributed less predictably than in non-fictional prose, while in non-fictional texts, the distribution of verbs, adjectives and prepositions is less predictable in comparison with fictional texts.

Visual inspection of the data in Table 2 and Table 3 does not *prima facie* show any clear patterns with respect to the differences in magnitude of the ApEn values (Table 2) and the ShEn values (Table 3), for each pair of columns. In order to determine whether the degrees of uncertainty observed for the various text category/feature combinations are a property of the texts as bags of boxes or as a function of the linear sequence of the boxes, we used classification tasks with a Support Vector Machine, which allows us to estimate the discriminatory power of each feature.

### 3.2. Classification

In two classification tasks, we determined what features can most efficiently classify or separate the categories of text under analysis—canonical vs. non-canonical fiction and fictional vs. non-fictional (expository) prose. We refer to the task of classifying canonical vs. non-canonical texts as ‘Task 1’ and the task of classifying non-fictional vs. fictional texts as ‘Task 2’. We used a Support Vector Machine (SVM) with a Radial Basis Function (RBF) kernel for the two tasks. As the categories to be classified are of different size, we used balanced accuracy as our evaluation measure. Wherever we compare classification results, we used the 5×2CV paired *t*-test [59] with a significance level of α=0.05. We report the mean of the 10 runs, 5 times 2-fold cross-validation, for each setting.

Table 4 shows the classification results for the two tasks using ApEn values and ShEn values calculated for each text as features for the classification task. As in Table 2 and Table 3, values that are significantly higher than their counterparts are highlighted with boldface. The top section of the table shows the results for each individual property. The most important observation is that ApEn separates canonical from non-canonical fictional texts better than ShEn does (Task 1).

Wherever the results are significantly better than random accuracy (50%), ApEn is more effective than ShEn. Moreover, for ApEn, classification is significantly different from random accuracy for all but one text property, i.e., Adverb, while the differences are not statistically significant for three text properties for ShEn, i.e., Sentence Length, Adjective and Adverb (indicated by a dagger^†^ in Table 4). Table 4 also shows the classification results when all text properties are taken into account. In Task 1, ApEn outperformed ShEn by a large margin (77.3% vs. 68.5%).

While the overall accuracy measures for ApEn may seem moderate in Task 1—77.3% using all features, with the POS-tag Noun alone reaching 73.6%—it should be borne in mind that this task is particularly difficult. Canonical and non-canonical texts belong to the same genre—fictional prose—and the differences between them can be expected to be subtle. The accuracy values for ShEn, which are significantly lower than those for ApEn, show that the difference between canonical and non-canonical fiction is not so much a matter of global (bag-of-boxes) distributions as it is a matter of sequential organization (sequence-of-boxes distribution).

The results for Task 2 differ strikingly from those for Task 1. Importantly, ShEn overall appeared to perform better than ApEn in this task. The results are significantly higher for Sentence Length, Noun and Verb. For three of the features—Adjective, Adverb and Pronoun— ApEn and ShEn values do not differ significantly. Concerning the results based on all features, the accuracy values of ShEn and ApEn are also similar, with values of >95%, and the observed difference is not significant. This result suggests that the differences between fictional and non-fictional texts are a matter of global distribution rather than sequential organization.

The right column of Table 4 shows another interesting result: The feature Pronoun alone classifies fictional vs. non-fictional texts with very high accuracy (≈95%), for both ApEn and ShEn. In fact, using all features does not lead to a significantly better performance than using Pronoun alone.

Given the prevalence of the feature Pronoun in the classification of fictional vs. non-fictional texts (Task 2), we repeated the task using all features except Pronoun, to gain a better understanding of the role of the remaining text properties. Without Pronoun, the performance of classification dropped to 89.7% and 91.0% for ApEn and ShEn, respectively, a considerable decrease for both features.

In comparison with other classification studies, the accuracy scores obtained in our study may appear to be rather moderate overall. Studies based on lexical material or *n*-grams may be more successful in text classification (see, for instance [60] on novels by Stephen King). We would like to emphasize, however, that we are interested in understanding the higher-level design features of texts, not their make-up in terms of low-level features, such as words or *n*-grams.

Our endeavour is thus more comparable to studies that aim to classify texts in terms of parameters associated with linguistic laws, such as Zipf’s law [34,35] and the Menzerath–Altmann law [61,62,63,64]. For comparison, we therefore ran classification tasks using parameters of these laws as input features (as suggested by a reviewer). The lambda-values of Zipfian distributions fitted to lemma counts delivered accuracy scores of 64.8% (Task 1) and 56.8% (Task 2). The two parameters *b* and *c* of a Menzerath–Altmann distribution ($y=ax^{b}e^{-cx}$) fitted to the average length of clauses and measured in tokens as a function of the number of clauses in a sentence, yielded accuracy scores of 55.8% (Task 1) and 68.6% (Task 2) (we used the package ‘menzerath’ for R [65] to extract the parameters with the function ‘menzerath()’ [method ‘MAL’]).

This illustrates, again, how difficult Task 1 is. Our experiments with the parameters of linguistic laws were only preliminary, and there are certainly ways of optimizing the classification process, e.g., by applying a more precise definition of ‘clause’ (we split sentences into clauses by relying on punctuation). In any case, they confirm that classification with a low number of features that describe a text as a whole is a difficult undertaking and that accuracy scores in the range of 75–80% as obtained with Approximate Entropy for Task 1 are less disappointing than they might appear to be on first sight. The lambda parameters of Zipf’s law and the two parameters of the Menzerath–Altmann law (*b* and *c*) are shown in Supplementary Figures S5 and S6, respectively.

### 3.3. Most Discriminative Features

As mentioned above, the discrimination of canonical vs. non-canonical texts (Task 1) is much more difficult than that of fictional vs. non-fictional texts (Task 2). While in Task 2 there is one prominent feature—Pronoun—the contributions of the features in Task 1 are more evenly distributed. In order to determine degrees of feature importance, we applied two methods.

First, we used sensitivity analysis [66]; the results are shown in Supplementary Figures S7 and S8. This analysis confirms the impression given by Table 4 that Noun and Verb are the most important discriminators for ApEn in Task 1, while Pronoun is the most important discriminator for ShEn. Second, we ran a brute-force search on the ApEn features as well as the ShEn ones (to give readers a visual impression of the discriminatory power of pairs of features, we provide pair plots of all features for ApEn and ShEn in the Supplementary Figures S1–S4).

**Figure 1:**
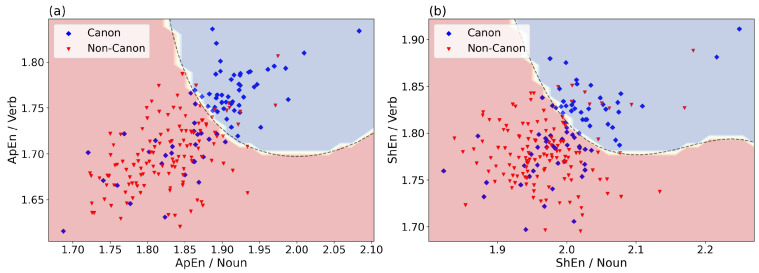
ApEn (**a**) and ShEn (**b**) of Noun and Verb, the two best features for classification of canonical vs. non-canonical texts (Task 1). ApEn and ShEn values of these two features provide an accuracy of 75.9% and 68.4%, respectively. The coloured regions and the border (dashed) line show the decision space of the Support Vector Machine.

Again, the most effective pair of ApEn features was that of Noun and Verb. Figure 1a visualizes the values of the two features for all fictional texts. The ApEn values of fictional texts in both the Noun and Verb series tended to be higher for canonical than for non-canonical texts. Moreover, the correlation between these two features, i.e., the ApEn of Noun and of Verb, was higher for canonical texts (Pearson coefficient 0.75) than for non-canonical texts (0.49). For comparison, Figure 1b shows the ShEn values for Noun and Verb. The figure demonstrates that the discriminative power of the two features is significantly lower than that of the corresponding ApEn values as shown in Figure 1a.

**Figure 2:**
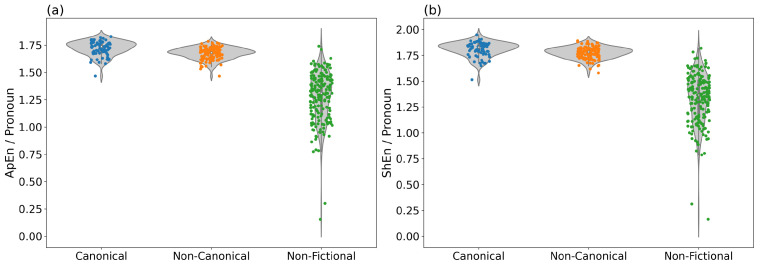
Values for ApEn (**a**) and ShEn (**b**) of Pronoun. These two features yield high accuracy for the classification of fictional vs. non-fictional texts (Task 2).

In Task 2, ApEn and ShEn of Pronoun were the most effective features in discriminating fictional from non-fictional texts with an accuracy of >95%. As Table 4 shows, adding more features does not improve the classification results significantly. In Figure 2a,b, the distributions of ApEn and ShEn values for Pronoun are visualized in the form of violin plots. The figures show that the values are clearly higher for fictional than for non-fictional texts, while the ranges of values for canonical and non-canonical texts largely overlap.

Note also that the values for non-fictional texts are very broadly distributed, while the values for fictional (canonical and non-canonical) texts are consistently very high. As there is hardly any difference between the plots for ApEn and ShEn, we can assume that the uncertainty due to the distribution of pronouns is a matter of global distribution, rather than sequential organization, as mentioned above.

## 4. Discussion and Conclusions

The most important result of our study can be summarized as follows: Canonical and non-canonical fictional texts differ in their degrees of predictability regarding the sequential distribution of the major parts of speech Noun, Pronoun, Verb, Adjective, Adverb and Preposition in windows of 25 tokens, and this was reflected in their higher Approximate Entropy (ApEn) values (cf. Table 2).

In other words, following a given window of text, there is less certainty about the frequency of specific parts of speech in the next window in canonical fictional texts, in comparison to non-canonical fictional texts. This result confirms our expectation that canonical fictional texts may be less predictable than non-canonical texts in terms of their textual structure. Whether or not this is perceived by a reader remains to be investigated. We assume that the observed differences are located at a medium level of text or discourse organization. They are probably not so much a matter of sentence-level syntax as they are of textual organization at the paragraph level.

Specifically, we suspect that frequency distributions of part-of-speech tags reflect discourse modes where the less predictable structural organization of canonical texts is due to (more unpredictable) shifts between discourse modes. The most important discourse modes in (traditional) fictional prose are those of narration and dialogue, followed perhaps by description. Verbs and nouns are important discriminators of discourse modes, insofar as verbs are prevalent in narration and dialogue, while nouns are more frequent in description and are particularly rare in dialogue.

In order to test this hypothesis, more detailed and thorough investigations will be needed. One way of approaching this task could be with Latent Dirichlet Allocation (LDA), which is commonly used for Topic Modelling [67]). If rhetorical modes are associated with multinomial distributions over parts of speech, as we assume, LDA-models (potentially supervised/labelled) could be trained on mixed-genre corpora. The models trained in this way could be used to assign to each window of text a distribution of discourse modes, and the resulting distributions could be analysed using methods like the ones applied in the present study, or other ways of capturing the global structural properties of texts (e.g., MFDFA [9]).

As the discriminative power of Shannon Entropy (ShEn) was lower than that of ApEn in Task 1, we assume that our results concerning the difference between canonical and non-canonical fictional texts do not reflect bag-of-boxes distributions but rather sequential organization within individual texts as reflected in sequences of boxes.

The results of our comparison between fictional and non-fictional prose were very different. The task of discriminating fictional from non-fictional texts was overall much easier than the classification of canonical vs. non-canonical fictional texts, as shown by the (balanced) accuracy scores of the classification tasks. This is not surprising, as we are here dealing with a question of genre classification, whereas canonical and non-canonical texts belong to the same genre and (by hypothesis) differ in terms of the textual structure.

Since ApEn did not fare better than ShEn in the fictional/non-fictional classification task, we assume that this is a matter of the bag-of-boxes distributions of text features, rather than of their sequential structure. Note also that there was no consistent pattern in the distribution of ShEn values across text properties. It appears that fictional and non-fictional texts differ in the ways parts of speech are distributed, with some of them showing flatter distributions (with higher entropy) and others showing steeper distributions (with lower entropy values) without a general trend.

An interesting observation that emerged from Task 2 was the central role of pronoun frequencies, which showed high performance. Pronouns are often not analysed in text classification and are often ignored as they are filtered out as stopwords. However, there are also studies acknowledging the importance of pronouns. For example, Kernot [68] showed that data taken from 30 articles written by three female and two male authors could be classified into gender categories by using only three pronouns, i.e., *my*, *her* and *its*.

Similarly, a study of sentimentalism in literature, Yu [69], found that pronouns are particularly valuable discriminators. In the context of register classification, the discriminatory power of pronouns is plausible. Qureshi et al. [70] found that the ratio of the number of adjectives to the number of pronouns is a good discriminator for distinguishing fictional from non-fictional texts.

Our finding that pronouns are informative when their predictability of occurrence is studied fits into this picture. While fictional texts are characterized by alternations between narrative passages and dialogue, the latter mode being associated with deictic pronouns (*I*, *you*), non-fictional prose can be expected to exhibit a more even distribution of anaphoric pronouns (*she*, *he*, *they*).

Our finding that the sequential structure of canonical texts is less predictable than that of non-canonical texts can be compared to results from vision studies. The basic perceptual features of visual images include, for example, oriented gradients of luminance or colour (edges). It has been shown that the distribution of edge orientations is less predictable across individual images of traditional artworks than in several types of non-art images [7]. In analogy to the present results for texts, the entropy of edge orientations is relatively high in visual artworks. High entropy of edge orientations can also be observed in other stimuli that beholders like more, including artificially generated visual patterns [71,72].

In the auditory domain, an intermediate degree of unpredictability and its resolution during listening are thought to evoke musical pleasure [36] in agreement with predictive coding accounts of brain function [37,73] (for a review of possible neural correlates of musical expectations in the human brain, see [74]). We speculate that a certain degree of unpredictability in the distribution of basic structural (perceptual) features is one of the hallmarks of aesthetically appreciated stimuli. Whether this hypothesis can be generalized to other types of text and whether this reflects domain-general perceptual and cognitive processes across sensory domains remains to be investigated.

## Figures and Tables

**Table 1:** Text categories in the Jena Expository and Fictional Prose (JEFP), version 2.0. The table shows, for each text category, the number of texts and the mean text length, measured in tokens, ± standard deviation.

Category	Number of Texts	Mean Length (×10^3^)
Canonical	76	199 ± 96
Non-Canonical	130	111 ± 56
Non-Fictional	185	171 ± 178

**Table 2:** Median values of Approximate Entropy (ApEn) for all text properties. ApEn values were analysed for two tasks: canonical (*N* = 76) vs. non-canonical (*N* = 130) texts and fictional (*N* = 206) vs. non-fictional (*N* = 185) texts. The asterisks indicate whether the differences between the two text categories of a given task are statistically significant (Mann–Whitney U test; ns, not significant; * p≤0.05; ** p≤0.01; and *** p≤0.001). Values that are significantly higher within a pair of columns are shown in boldface. 95% confidence intervals for the median (according to [58]) are shown in parentheses.

Text Property	Canonical	Non-Canonical	Fictional	Non-Fictional
Sentence Length	1.86 (1.83, 1.89)	1.87 (1.86, 1.90) ${}^{\mathrm{ns}}$	1.87 (1.86, 1.88)	1.90 (1.88, 1.92) ${}^{\mathrm{ns}}$
Noun	**1.89 (1.88, 1.91)**	1.83 (1.81, 1.84) ***	**1.85 (1.84, 1.86)**	1.82 (1.81, 1.84) **
Verb	**1.75 (1.73, 1.76)**	1.70 (1.69, 1.71) ***	1.714 (1.706, 1.723)	**1.756 (1.745, 1.764)** ***
Adjective	**1.50 (1.49, 1.52)**	1.45 (1.43, 1.48) ***	1.488 (1.469, 1.494)	**1.58 (1.55, 1.60)** ***
Adverb	**1.51 (1.49, 1.53)**	1.48 (1.46, 1.49) **	**1.49 (1.48, 1.50)**	1.36 (1.34, 1.39) ***
Pronoun	**1.74 (1.71, 1.76)**	1.681 (1.675, 1.691) ***	**1.695 (1.685, 1.704)**	1.31 (1.28, 1.36) ***
Preposition	**1.71 (1.70, 1.72)**	1.67 (1.66, 1.68) ***	1.678 (1.672, 1.683)	**1.691 (1.686, 1.697)** ***

**Table 3:** Median values of Shannon Entropy (ShEn) for all text properties. ApEn values were analysed for two tasks: canonical (*N* = 76) vs. non-canonical (*N* = 130) texts and fictional (*N* = 206) vs. non-fictional (*N* = 185) texts. The asterisks indicate whether the differences between the two text categories of a given task are statistically significant (Mann–Whitney U test; ns, not significant; * p≤0.05; ** p≤0.01; and *** p≤0.001). Values that are significantly higher within a pair of columns are shown in boldface. 95% confidence intervals for the median (according to [58]) are shown in parentheses.

Text Property	Canonical	Non-Canonical	Fictional	Non-Fictional
Sentence Length	3.96 (3.88, 4.05)	3.96 (3.87, 4.08) ${}^{\mathrm{ns}}$	3.96 (3.91, 4.03)	**4.10 (4.07, 4.16)** ***
Noun	**2.00 (1.99, 2.02)**	1.97 (1.95, 1.98) ***	1.98 (1.97, 1.99)	1.97 (1.95, 1.99) ${}^{\mathrm{ns}}$
Verb	**1.80 (1.79, 1.81)**	1.777 (1.772, 1.783) ***	1.785 (1.779, 1.792)	**1.844 (1.836, 1.853)** ***
Adjective	**1.54 (1.53, 1.55)**	1.49 (1.47, 1.53) ***	1.52 (1.51, 1.53)	**1.63 (1.61, 1.66)** ***
Adverb	**1.54 (1.51, 1.55)**	1.51 (1.49, 1.53) *	**1.52 (1.51, 1.53)**	1.40 (1.37, 1.42) ***
Pronoun	**1.83 (1.80, 1.84)**	1.78 (1.77, 1.79) ***	**1.79 (1.78, 1.80)**	1.37 (1.33, 1.42) ***
Preposition	**1.75 (1.74, 1.77)**	1.73 (1.72, 1.74) ***	1.736 (1.729, 1.744)	**1.76 (1.75, 1.77)** ***

**Table 4:** Balanced accuracy of classification (in %) for the single features for the canonical/non-canonical distinction (Task 1) and the non-fictional/fictional distinction (Task 2). To compare classification results, we used the 5 × 2CV paired *t*-test [59] with a significance level of α=0.05. Values that are significantly higher within a pair of columns are shown in boldface. All values are significantly different (*p*≤ 0.05) from random accuracy (50%), except where indicated by a dagger (†).

	Task 1		Task 2	
	ApEn	ShEn	ApEn	ShEn
Sentence Length	**54.0 ± 1.6**	50.0 ± 1.0 †	53.6 ± 2.9	**61.7 ± 2.3**
Noun	**73.6 ± 2.9**	60.0 ± 4.5	57.4 ± 1.9	**64.2 ± 1.8**
Verb	**71.3 ± 3.4**	56.2 ± 3.8	65.5 ± 2.4	**74.0 ± 1.6**
Adjective	**55.2 ± 2.5**	51.5 ± 2.7 †	71.7 ± 2.1	74.3 ± 1.0
Adverb	51.6 ± 1.4 †	51.0 ± 1.5 †	72.8 ± 2.2	73.0 ± 2.9
Pronoun	**68.0 ± 1.7**	63.8 ± 1.8	95.1 ± 1.5	95.0 ± 1.7
Preposition	**69.1 ± 2.4**	59.7 ± 1.7	56.9 ± 2.6	**61.4 ± 1.3**
All	**77.3 ± 2.6**	68.5 ± 2.3	95.4 ± 1.8	96.5 ± 1.9

## Data Availability

The data presented in this study are available on request from the corresponding author. The data are not publicly available due to copyright restrictions.

## Supplementary Materials

The following supporting information can be downloaded at: https://www.mdpi.com/article/10.3390/e24020278/s1: Table S1: List of texts in the Jena Corpus of Expository and Fictional Prose (JEFP Corpus, Version 2.0). Figure S1: Pair-plot of all Approximate Entropy (ApEn) features in fictional/canonical and fictional/non-canonical texts. Figure S2: Pair-plot of all Shannon Entropy (ShEn) features in fictional/canonical and fictional/non-canonical texts. Figure S3: Pair-plot of all Approximate Entropy (ApEn) features in fictional/canonical, fictional/non-canonical and non-fictional texts. Figure S4: Pair-plot of all Shannon Entropy (ShEn) features in fictional/canonical, fictional/non-canonical and non-fictional texts. Figure S5: Zipf’s law coefficient (lambda) of fictional/canonical, fictional/non-canonical and non-fictional texts. Figure S6: The two parameters (*b* and *c*) of the Menzerath–Altmann law in fictional/canonical, fictional/non-canonical and non-fictional texts. Figure S7: Sensitivity analysis of ApEn features and ShEn features in classification of fictional/canonical and fictional/non-canonical texts. Figure S8: Sensitivity analysis of ApEn features and ShEn features in classification of fictional and non-fictional texts.

## References

[1] Craig H., Kinney A.F. (2009). Shakespeare, Computers, and the Mystery of Authorship.

[2] Koppel M., Schler J., Argamon S. (2009). Computational Methods in Authorship Attribution. J. Am. Soc. Inf. Sci. Technol..

[3] Biber D. (1995). Dimensions of Register Variation. A Cross-linguistic Comparison.

[4] Lee D. (2001). Genres, Registers, Text Types, Domains and Styles: Clarifying the Concepts and Navigating a Path through the BNC Jungle. Technology.

[5] Fechner G.T. (1876). Vorschule der Ästhetik.

[6] Bell C. (1914). Art.

[7] Redies C., Brachmann A., Wagemans J. (2017). High Entropy of Edge Orientations Characterizes Visual Artworks From Diverse Cultural Backgrounds. Vis. Res..

[8] Brachmann A., Redies C. (2017). Computational and Experimental Approaches to Visual Aesthetics. Front. Comput. Neurosci..

[9] Mohseni M., Gast V., Redies C. (2021). Fractality and Variability in Canonical and Non-Canonical English Fiction and in Non-Fictional Texts. Front. Psychol..

[10] Diessel H. (2019). The Grammar Network. How Linguistic Structure is Shaped by Language Use.

[11] Hartung F., Wang Y., Mak M., Willems R., Chatterjee A. (2021). Aesthetic Appraisals of Literary Style and Emotional Intensity in Narrative Engagement Are Neurally Dissociable. Commun. Biol..

[12] Simonton D.K. (1990). Lexical Choices and Aesthetic Success: A Computer Content Analysis of 154 Shakespeare Sonnets. Comput. Humanit..

[13] Forsyth R.S. (2000). Pops and Flops: Some Properties of Famous English Poems. Empir. Stud. Arts.

[14] Kao J., Jurafsky D. A Computational Analysis of Style, Affect, and Imagery in Contemporary Poetry. Proceedings of the NAACL-HLT 2012 Workshop on Computational Linguistics for Literature.

[15] Ashok V., Feng S., Choi Y. Success With Style: Using Writing Style to Predict the Success of Novels. Proceedings of the 2013 Conference on Empirical Methods in Natural Language Processing.

[16] Maharjan S., Arevalo J., Montes M., González F., Solorio T. (2017). A Multi-task Approach to Predict Likability of Books. Proceedings of the 15th Conference of the European Chapter of the Association for Computational Linguistics: Volume 1, Long Papers.

[17] Maharjan S., Kar S., Montes M., González F.A., Solorio T. (2018). Letting Emotions Flow: Success Prediction by Modeling the Flow of Emotions in Books. Proceedings of the 2018 Conference of the North American Chapter of the Association for Computational Linguistics: Human Language Technologies, Volume 2 (Short Papers).

[18] Montemurro M.A., Zanette D.H. (2011). Universal Entropy of Word Ordering Across Linguistic Families. PLoS ONE.

[19] Montemurro M.A., Zanette D.H., Degli Esposti M., Altmann E.G., Pachet F. (2016). Complexity and Universality in the Long-Range Order of Words. Creativity and Universality in Language.

[20] Futrell R., Mahowald K., Gibson E. (2015). Quantifying Word Order Freedom in Dependency Corpora. Proceedings of the Third International Conference on Dependency Linguistics.

[21] Koplenig A., Meyer P., Wolfer S., Müller-Spitzer C. (2017). The Statistical Trade-off Between Word Order and Word Structure—Large-Scale Evidence for the Principle of Least Effort. PLoS ONE.

[22] Piantadosi S.T., Tily H., Gibson E. (2011). Word Lengths Are Optimized for Efficient Communication. Proc. Natl. Acad. Sci. USA.

[23] Mahowald K., Fedorenko E., Piantadosi S.T., Gibson E. (2013). Info/Information Theory: Speakers Choose Shorter Words in Predictive Contexts. Cognition.

[24] Ferrer-i-Cancho R., Bentz C., Seguin C. (2015). Compression and the Origins of Zipf’s Law of Abbreviation. arXiv.

[25] Kanwal J., Smith K., Culbertson J., Kirby S. (2017). Zipf’s Law of Abbreviation and the Principle of Least Effort: Language users optimise a miniature lexicon for efficient communication. Cognition.

[26] Bentz C., Verkerk A., Kiela D., Hill F., Buttery P. (2015). Adaptive Communication: Languages with More Non-Native Speakers Tend to Have Fewer Word Forms. PLoS ONE.

[27] Kalimeri M., Constantoudis V., Papadimitriou C., Karamanos K., Diakonos F.K., Papageorgiou H. (2015). Word-length Entropies and Correlations of Natural Language Written Texts. J. Quant. Linguist..

[28] Ehret K., Szmrecsanyi B., Baechler R., Seiler G. (2016). An Information-Theoretic Approach to Assess Linguistic Complexity. Complexity, Isolation, and Variation.

[29] Hernández-Gómez C., Basurto-Flores R., Obregón-Quintana B., Guzmán-Vargas L. (2017). Evaluating the Irregularity of Natural Languages. Entropy.

[30] Bentz C., Alikaniotis D., Cysouw M., Ferrer-i Cancho R. (2017). The Entropy of Words—Learnability and Expressivity across More than 1000 Languages. Entropy.

[31] Febres G., Jaffe K. (2017). Quantifying Structure Differences in Literature Using Symbolic Diversity and Entropy Criteria. J. Quant. Linguist..

[32] Chang M.C., Yang A.C.C., Stanley H.E., Peng C.K. (2017). Measuring Information-Based Energy and Temperature of Literary Texts. Phys. A Stat. Mech. Appl..

[33] Drożdż S., Oświȩcimka P., Kulig A., Kwapień J., Bazarnik K., Grabska-Gradzińska I., Rybicki J., Stanuszek M. (2016). Quantifying Origin and Character of Long-Range Correlations in Narrative Texts. Inf. Sci..

[34] Zipf G.K. (1949). Human Behavior and the Principle of Least Effort.

[35] Ferrer i Cancho R., Solé R. (2003). Least Effort and the Origins of Scaling in Human Language. Proc. Natl. Acad. Sci. USA.

[36] Gold B.P., Pearce M.T., Mas-Herrero E., Dagher A., Zatorre R.J. (2019). Predictability and Uncertainty in the Pleasure of Music: A Reward for Learning?. J. Neurosci..

[37] Koelsch S., Vuust P., Friston K. (2019). Predictive Processes and the Peculiar Case of Music. Trends Cogn. Sci..

[38] Guillory J. (1987). Canonical and Non-canonical: A Critique of the Current Debate. ELH.

[39] Even-Zohar I. (1990). Polysystem Studies. Poet. Today.

[40] Underwood T., Sellers J. (2016). The Long Durée of Literary Prestige. Mod. Lang. Q..

[41] Gerlach M., Font-Clos F. (2020). A Standardized Project Gutenberg Corpus for Statistical Analysis of Natural Language and Quantitative Linguistics. Entropy.

[42] Green C. (2017). Introducing the Corpus of the Canon of Western Literature: A Corpus for Culturomics and Stylistics. Lang. Lit..

[43] Bloom H. (1994). The Western Canon: The Books and School of the Ages.

[44] Reagan A.J., Mitchell L., Kiley D., Danforth C.M., Dodds P.S. (2016). The Emotional Arcs of Stories Are Dominated by Six Basic Shapes. EPJ Data Sci..

[45] Qi P., Zhang Y., Zhang Y., Bolton J., Manning C.D. (2020). Stanza: A Python Natural Language Processing Toolkit for Many Human Languages. Proceedings of the 58th Annual Meeting of the Association for Computational Linguistics: System Demonstrations.

[46] Smith C. (2003). Modes of Discourse. The Local Structure of Texts.

[47] Eisen M., Ribeiro A., Segarra S., Egan G. (2017). Stylometric analysis of Early Modern period English plays. Digit. Scholarsh. Humanit..

[48] Segarra S., Eisen M., Ribeiro A. (2015). Authorship Attribution Through Function Word Adjacency Networks. IEEE Trans. Signal Process..

[49] Brown P., Eisen M., Segarra S., Ribeiro A., Egan G. (2021). How the Word Adjacency Network Algorithm Works. Digit. Scholarsh. Humanit..

[50] Pincus S.M. (1991). Approximate Entropy as a Measure of System Complexity. Proc. Natl. Acad. Sci. USA.

[51] Li X., Cui S., Voss L. (2008). Using Permutation Entropy to Measure the Electroencephalographic Effects of Sevoflurane. Anesthesiology.

[52] Hayashi K., Shigemi K., Sawa T. (2012). Neonatal Electroencephalography Shows Low Sensitivity to Anesthesia. Neurosci. Lett..

[53] Lee G., Fattinger S., Mouthon A.L., Noirhomme Q., Huber R. (2013). Electroencephalogram Approximate Entropy Influenced by Both Age and Sleep. Front. Neuroinformatics.

[54] Richman J., Moorman J. (2000). Physiological Time-Series Analysis Using Approximate Entropy and Sample Entropy. Am. J. Physiol. Heart Circ. Physiol..

[55] Costa M., Goldberger A.L., Peng C.K. (2005). Multiscale Entropy Analysis of Biological Signals. Phys. Rev. E.

[56] Ahmed M., Mandic D. (2011). Multivariate Multiscale Entropy: A Tool for Complexity Analysis of Multichannel Data. Phys. Rev. Stat. Nonlinear Soft Matter Phys..

[57] Makowski D., Pham T., Lau Z.J., Brammer J.C., Lespinasse F., Pham H., Schölzel C., Chen S.H.A. (2021). NeuroKit2: A Python Toolbox for Neurophysiological Signal Processing. Behav. Res. Methods.

[58] Zar J.H. (2010). Biostatistical Analysis, 5 ed..

[59] Dietterich T.G. (1998). Approximate Statistical Tests for Comparing Supervised Classification Learning Algorithms. Neural Comput..

[60] van Cranenburgh A., Ketzan E. (2021). Stylometric Literariness Classification: The Case of Stephen King. Proceedings of the 5th Joint SIGHUM Workshop on Computational Linguistics for Cultural Heritage, Social Sciences, Humanities and Literature.

[61] Menzerath P., de Oleza J. (1928). Spanische Lautdauer. Eine experimentelle Untersuchung.

[62] Menzerath P. (1954). Die Architektonik des deutschen Wortschatzes.

[63] Altmann G. (1980). Prolegomena to Menzerath’s law. Glottometrika.

[64] Semple S., i Cancho R.F., Gustison M.L. (2020). Linguistic laws in biology. Trends Ecol. Evol..

[65] Sellis D. (2022). menzerath: Explore Data Following The Menzerath–Altmann Law. R Package Version 0.1.2. http://cran.r-project.org/web/packages/mvnfast/vignettes/mvnfast.html.

[66] Cortez P., Embrechts M.J. (2013). Using Sensitivity Analysis and Visualization Techniques to Open Black Box Data Mining Models. Inf. Sci..

[67] Blei D.M., Ng A.Y., Jordan M.I. (2003). Latent Dirichlet Allocation. J. Mach. Learn. Res..

[68] Kernot D. (2018). Can Three Pronouns Discriminate Identity in Writing?. Data and Decision Sciences in Action.

[69] Yu B. (2008). An Evaluation of Text Classification Methods for Literary Study. Lit. Linguist. Comput..

[70] Qureshi M.R., Ranjan S., Rajkumar R., Shah K. (2019). A Simple Approach to Classify Fictional and Non-Fictional Genres. Proceedings of the Second Workshop on Storytelling.

[71] Grebenkina M., Brachmann A., Bertamini M., Kaduhm A., Redies C. (2018). Edge-Orientation Entropy Predicts Preference for Diverse Types of Man-Made Images. Front. Neurosci..

[72] Stanischewski S., Altmann C.S., Brachmann A., Redies C. (2020). Aesthetic Perception of Line Patterns: Effect of Edge-Orientation Entropy and Curvilinear Shape. i-Perception.

[73] Kraus N. (2020). The Joyful Reduction of Uncertainty: Music Perception as a Window to Predictive Neuronal Processing. J. Neurosci..

[74] Salimpoor V.N., Zald D.H., Zatorre R.J., Dagher A., McIntosh A.R. (2015). Predictions and the Brain: How Musical Sounds Become Rewarding. Trends Cogn. Sci..

