# Supplementary material for: Approximate Entropy in Canonical and Non-Canonical Fiction
Source: Entropy (Basel). 2022 Feb 15;24(2):278. doi: 10.3390/e24020278 (PMC8870941; doi:10.3390/e24020278)
Supplement: Supplementary file 1 [file entropy-24-00278-s001.zip › entropy-1574097-supplementary.pdf]

Supplementary Materials: Approximate Entropy in Canonical and Non-Canonical Fiction

Table S1: List of texts in the Jena Corpus of Expository and Fictional Prose (JEFP Corpus), Version 2.0. Canonical texts were selected from the Corpus of Canonical Western Literature. Non-canonical texts were downloaded from www.smashwords.com, www.goodreads.com, www.feedbooks.com, or Project Gutenberg. Non-fictional texts were sampled from Project Gutenberg.

	Title	Author(s)	Year of Publication	Category
1	Little Dorrit	Charles Dickens	1857	Canonical
2	Oliver Twist	Charles Dickens	1839	Canonical
3	The Life and Adventures of Nicholas Nickleby	Charles Dickens	1839	Canonical
4	The Mystery of Edwin Drood	Charles Dickens	1870	Canonical
5	The Pickwick Papers	Charles Dickens	1836	Canonical
6	Jane Eyre	Charlotte Bronte	1847	Canonical
7	Villette	Charlotte Bronte	1853	Canonical
8	Cranford	Elizabeth Gaskell	1853	Canonical
9	Mary Barton	Elizabeth Gaskell	1848	Canonical
10	North and South	Elizabeth Gaskell	1854	Canonical
11	Agnes Grey	Anne Bronte	1847	Canonical
12	Adam Bede	George Eliot	1859	Canonical
13	Daniel Deronda	George Eliot	1876	Canonical
14	Middlemarch	George Eliot	1872	Canonical
15	Silas Marner	George Eliot	1861	Canonical
16	The Mill on the Floss	George Eliot	1860	Canonical
17	Emma	Jane Austen	1815	Canonical
18	Mansfield Park	Jane Austen	1814	Canonical
19	Persuasion	Jane Austen	1818	Canonical
20	Pride and Prejudice	Jane Austen	1813	Canonical
21	The Picture of Dorian Gray	Oscar Wilde	1890	Canonical
22	The Tenant of Wildfell Hall	Anne Bronte	1848	Canonical
23	Sartor Resartus	Thomas Carlyle	1834	Canonical
24	Old Mortality	Walter Scott	1816	Canonical
25	Redgauntlet	Walter Scott	1824	Canonical
26	The Heart of Midlothian	Walter Scott	1818	Canonical
27	Waverley	Walter Scott	1814	Canonical
28	No Name	Wilkie Collins	1862	Canonical
29	The Moonstone	Wilkie Collins	1868	Canonical
30	The Woman in White	Wilkie Collins	1859	Canonical
31	The History of Henry Esmond	William Makepeace Thackeray	1852	Canonical
32	Vanity Fair	William Makepeace Thackeray	1847	Canonical
33	Dracula	Bram Stoker	1897	Canonical
34	The Well at the World's end	William Morris	1896	Canonical
35	The Narrative of Arthur Gordon Pym	Edgar Allan Poe	1838	Canonical
36	The Ambassadors	Henry James	1903	Canonical
37	The Awkward Age	Henry James	1899	Canonical
38	The Bostonians	Henry James	1886	Canonical
39	The Golden Bowl	Henry James	1904	Canonical
40	The Portrait of a Lady	Henry James	1881	Canonical
41	The Wings of Dove	Henry James	1902	Canonical
42	Moby Dick	Herman Melville	1851	Canonical
43	The Deerslayers	James Fenimore Cooper	1841	Canonical
44	A Christmas Carol	Charles Dickens	1843	Canonical
45	Little Women	Louisa May Alcott	1868	Canonical
46	Puddnhead Wilson	Mark Twain	1893	Canonical
47	The Adventures of Finn	Mark Twain	1884	Canonical
48	The Mysterious Stranger	Mark Twain	1916	Canonical
49	The Marble Faun	Nathaniel Hawthorne	1859	Canonical
50	The Scarlet Letter	Nathaniel Hawthorne	1850	Canonical
51	Walden	Henry David Thoreau	1854	Canonical
52	A Conneticut Yankee in King Arthurs	Mark Twain	1889	Canonical
53	Babbitt	Sinclair Lewis	1922	Canonical
54	A Tale of Two Cities	Charles Dickens	1859	Canonical

Continued on next page

Table S1 – *Continued from previous page*

	Title	Author(s)	Year of Publication	Category
55	Sister Carrie	Theodore Dreiser	1900	Canonical
56	My Antonia	Willa Cather	1918	Canonical
57	The Old Wives Tale	Arnold Bennett	1908	Canonical
58	Portrait of the Artist as a Young Man	James Joyce	1916	Canonical
59	Ulysses	James Joyce	1922	Canonical
60	Lord Jim	Joseph Conrad	1900	Canonical
61	Nostromo	Joseph Conrad	1904	Canonical
62	The Secret Agent	Joseph Conrad	1907	Canonical
63	Under Western Eyes	Joseph Conrad	1911	Canonical
64	Victory: An Island Tale	Joseph Conrad	1915	Canonical
65	Bleak House	Charles Dickens	1853	Canonical
66	The Rainbow	D. H. Lawrence	1915	Canonical
67	Women in Love	D. H. Lawrence	1920	Canonical
68	Kim	Rudyard Kipling	1901	Canonical
69	Puck of Pooks Hill	Rudyard Kipling	1906	Canonical
70	Jude the Obscure	Thomas Hardy	1895	Canonical
71	Tess of the dUrbervilles	Thomas Hardy	1891	Canonical
72	The Mayor of Casterbridge	Thomas Hardy	1886	Canonical
73	The Return of the Native	Thomas Hardy	1878	Canonical
74	David Copperfield	Charles Dickens	1850	Canonical
75	Great Expectations	Charles Dickens	1860	Canonical
76	Hard Times	Charles Dickens	1854	Canonical
77	A Prisoner in Fairyland	Algernon Blackwood	1913	Non-Canonical
78	The Centaur	Algernon Blackwood	1911	Non-Canonical
79	Ruth Fielding at the War Front	Alice B. Emerson	1918	Non-Canonical
80	The International Spy	Allen Upward	1904	Non-Canonical
81	A Texas Matchmaker	Andy Adams	1904	Non-Canonical
82	The Filigree Ball	Anna Katharine Green	1903	Non-Canonical
83	Looking Further Backward	Arthur Dudley Vinton	1890	Non-Canonical
84	The Hill Of Dreams	Arthur Machen	1907	Non-Canonical
85	Jean of the Lazy A	Burton E. Stevenson	1915	Non-Canonical
86	The Gloved Hand	Baroness Emma Orczy	1913	Non-Canonical
87	The Filibusters	Charles John Cutcliffe Wright Hyne	1900	Non-Canonical
88	Wunpost	Dane Coolidge	1920	Non-Canonical
89	Love Insurance	Earl Derr Biggers	1914	Non-Canonical
90	The Wouldbegoods	Edith Nesbit	1899	Non-Canonical
91	Wet Magic	Edith Nesbit	1913	Non-Canonical
92	An Amiable Charlatan	Edward Phillips Oppenheim	1916	Non-Canonical
93	The Double Traitor	Edward Phillips Oppenheim	1915	Non-Canonical
94	The Zeppelin 's Passenger	Edward Phillips Oppenheim	1918	Non-Canonical
95	The People of the Ruins	Edward Shanks	1920	Non-Canonical
96	The Honor of the Name	Ernest Bramah Smith	1891	Non-Canonical
97	The Riddle of the Sands	Eugene Percy Lyle	1903	Non-Canonical
98	The Missourian	Ford Madox Ford	1905	Non-Canonical
99	Privy Seal	Frederic Arnold Kummer	1907	Non-Canonical
100	Condemned as a Nihilist	Harold MacGrath	1893	Non-Canonical
101	The Afterglow	George Allan England	1913	Non-Canonical
102	The Flying Legion	George Allan England	1920	Non-Canonical
103	West Wind Drift	George Barr McCutcheon	1920	Non-Canonical
104	Trilby	George W. Ogden	1894	Non-Canonical
105	Olga Romanoff or , The Syren of the Skies	George F. Worts	1894	Non-Canonical
106	The Princess and Curdie	George Griffith	1883	Non-Canonical
107	The Adventures of Don Lavington	George MacDonald	1896	Non-Canonical
108	A Voyage to the Moon	George Manville Fenn	1827	Non-Canonical
109	Man on the Box	Harold MacGrath	1904	Non-Canonical
110	The Puppet Crown	Homer Eon Flint	1901	Non-Canonical
111	Men of Iron	Ida Alexa Ross Wylie	1891	Non-Canonical
112	Towards Morning	James Branch Cabell	1918	Non-Canonical
113	A Strange Manuscript Found in a Copper Cylinder	James De Mille	1888	Non-Canonical
114	Lost in the Fog	James Malcom Rymer	1870	Non-Canonical
115	Varney the Vampire	James Oliver Curwood	1847	Non-Canonical
116	The Danger Trail	John Meade Falkner	1910	Non-Canonical

Continued on next page

Table S1 – *Continued from previous page*

	Title	Author(s)	Year of Publication	Category
117	The Lost Stradivarius	John Meade Falkner	1895	Non-Canonical
118	The Nebuly Coat	Joseph Hocking	1903	Non-Canonical
119	The Weapons of Mystery	Joseph Smith Fletcher	1890	Non-Canonical
120	Diane of the Green Van	Lord Dunsany	1914	Non-Canonical
121	The Treasure Trail	Mary E. Bradley	1918	Non-Canonical
122	Mizora : A Prophecy	Mary Roberts Rinehart	1889	Non-Canonical
123	Across the Zodiac	Percy Greg	1880	Non-Canonical
124	Bardelys the Magnificent	Rafael Sabatini	1905	Non-Canonical
125	Soldiers of Fortune	Richard Harding Davis	1897	Non-Canonical
126	The Beetle	Richard Marsh	1897	Non-Canonical
127	The Triumphs of Eugne Valmont	Robert Barr	1906	Non-Canonical
128	Erling the Bold	Robert Michael Ballantyne	1869	Non-Canonical
129	The Dog Crusoe and His Master	Robert Michael Ballantyne	1894	Non-Canonical
130	Ailsa Paige	Robert William Chambers	1910	Non-Canonical
131	In Search of the Unknown	Robert William Chambers	1904	Non-Canonical
132	In the Quarter	Robert William Chambers	1894	Non-Canonical
133	Erewhon , or Over The Range	Samuel Butler	1910	Non-Canonical
134	The road to Frontenac	Samuel Merwin	1901	Non-Canonical
135	The Revolt of Man	Sir Walter Besant	1882	Non-Canonical
136	The Brass Bottle	Thomas Anstey Guthrie	1900	Non-Canonical
137	The Doomsman	Van Tassel Sutphen	1906	Non-Canonical
138	The Border Legion	Zane Grey	1916	Non-Canonical
139	The Daltons; Or, Three Roads In Life. Volume I (of II)	Charles James Lever	1850	Non-Canonical
140	Melmoth the Wanderer, Vol. 3	Charles Robert Maturin	1820	Non-Canonical
141	Melmoth the Wanderer, Vol. 2	Charles Robert Maturin	1820	Non-Canonical
142	The Wanderer; or, Female Difficulties (Volume 4 of 5)	Fanny Burney	1814	Non-Canonical
143	The Wanderer; or, Female Difficulties (Volume 2 of 5)	Fanny Burney	1814	Non-Canonical
144	The Wanderer; or, Female Difficulties (Volume 3 of 5)	Fanny Burney	1814	Non-Canonical
145	The Vicar of Wrexhill	Frances Milton Trollope	1837	Non-Canonical
146	The Fortunes of the Colville Family; or, A Cloud with its Silver Lining	Frank Edward Smedley	1853	Non-Canonical
147	Harry Coverdale's Courtship, and All That Came of It	Frank Edward Smedley	1854	Non-Canonical
148	Lewis Arundel; Or, The Railroad Of Life	Frank Edward Smedley	1852	Non-Canonical
149	The Little Savage	Frederick Marryat	1848	Non-Canonical
150	Newton Forster	Frederick Marryat	1832	Non-Canonical
151	Snarleyyow; or, The Dog Fiend	Frederick Marryat	1837	Non-Canonical
152	Travels and Adventures of Monsieur Violet	Frederick Marryat	1843	Non-Canonical
153	The Privateer's-Man, One hundred Years Ago	Frederick Marryat	1846	Non-Canonical
154	The Little Savage	Frederick Marryat	1848	Non-Canonical
155	Snarleyyow, or, the Dog Fiend	Frederick Marryat	1837	Non-Canonical
156	Newton Forster; Or, The Merchant Service	Frederick Marryat	1832	Non-Canonical
157	Mr. Midshipman Easy	Frederick Marryat	1836	Non-Canonical
158	Arrah Neil; or, Times of Old	George Payne Rainsford James	1843	Non-Canonical
159	The Castle of Ehrenstein: Its Lords Spiritual and Temporal; Its Inhabitants Earthly and Unearthly	George Payne Rainsford James	1847	Non-Canonical
160	Forest Days: A Romance of Old Times	George Payne Rainsford James	1843	Non-Canonical
161	A Voyage to the Moon: With Some Account of the Manners and Customs, Science and Philosophy, of the People of Morosofia, and Other Lunarians	George Tucker	1827	Non-Canonical
162	Market Harborough, and Inside the Bar	George John Whyte-Melville	1858	Non-Canonical
163	The Gladiators. A Tale of Rome and Judæa	George John Whyte-Melville	1863	Non-Canonical
164	The Mother's Recompense, Volume 2: A Sequel to Home Influence	Grace Aguilar	1874	Non-Canonical
165	The Mother's Recompense, Volume 1: A Sequel to Home Influence	Grace Aguilar	1874	Non-Canonical
166	The Vale of Cedars; Or, The Martyr	Grace Aguilar	1850	Non-Canonical
167	Jasper Lyle	Mary Augusta Ward	1851	Non-Canonical
168	The Eskdale Herd-boy: A Scottish Tale for the Instruction and Amusement of Young People	Martha Blackford	1819	Non-Canonical
169	Mary Erskine	Jacob Abbott	1850	Non-Canonical
170	Bruno; or, lessons of fidelity, patience, and self-denial taught by a dog	Jacob Abbott	1854	Non-Canonical
171	Rollo in Rome	Jacob Abbott	1858	Non-Canonical

Continued on next page

Table S1 – *Continued from previous page*

	Title	Author(s)	Year of Publication	Category
172	Rollo in London	Jacob Abbott	1850	Non-Canonical
173	Among the Brigands	James De Mille	1871	Non-Canonical
174	The Lily and the Cross: A Tale of Acadia	James De Mille	1875	Non-Canonical
175	Fire in the Woods: Illustrated	James De Mille	1872	Non-Canonical
176	Cord and Creese	James De Mille	1869	Non-Canonical
177	The Three Perils of Man; or, War, Women, and Witchcraft, Vol. 3 (of 3)	James Hogg	1822	Non-Canonical
178	The Three Perils of Man; or, War, Women, and Witchcraft, Vol. 2 (of 3)	James Hogg	1822	Non-Canonical
179	The Provost	John Galt	1822	Non-Canonical
180	Ringan Gilhaize, or, The Covenanters	John Galt	1823	Non-Canonical
181	Valerius. A Roman Story	John Gibson Lockhart	1821	Non-Canonical
182	The Manoeuvring Mother (vol. 3 of 3)	Lady Charlotte Susan Maria Bury	1842	Non-Canonical
183	The Manoeuvring Mother (vol. 2 of 3)	Lady Charlotte Susan Maria Bury	1842	Non-Canonical
184	The Manoeuvring Mother (vol. 1 of 3)	Lady Charlotte Susan Maria Bury	1842	Non-Canonical
185	The Annals of the Poor	Legh Richmond	1814	Non-Canonical
186	Aunt Kitty's Tales	Maria Jane McIntosh	1847	Non-Canonical
187	Camperdown; or, News from our neighbourhood	Mary Griffith	1836	Non-Canonical
188	The Actress' Daughter: A Novel	May Agnes Fleming	1879	Non-Canonical
189	Emilie the Peacemaker	Mrs. Thomas Geldart	1851	Non-Canonical
190	The Old Church Clock	Richard Parkinson	1843	Non-Canonical
191	Sheppard Lee, Written by Himself. Vol. 1 (of 2)	Robert Montgomery Bird	1836	Non-Canonical
192	The Young Trail Hunters: Or, the Wild Riders of the Plains. The Veritable Adventures of Hal Hyde and Ned Brown, on Their Journey Across the Great Plains of the South-West	Samuel Woodworth Cozzens	1876	Non-Canonical
193	Watch—Work—Wait: Or, The Orphan's Victory	Sarah Ann Myers	1859	Non-Canonical
194	Pine Needles	Susan Bogert Warner	1877	Non-Canonical
195	Confession; Or, The Blind Heart. A Domestic Story	William Gilmore Simms	1841	Non-Canonical
196	Antony Waymouth; Or, The Gentlemen Adventurers	William Henry Giles Kingston	1865	Non-Canonical
197	Clara Maynard; Or, The True and the False: A Tale of the Times	William Henry Giles Kingston	1877	Non-Canonical
198	The Story of Nelson: also "The Grateful Indian", "The Boatswain's Son"	William Henry Giles Kingston	1860	Non-Canonical
199	Off to Sea: The Adventures of Jovial Jack Junker on his Road to Fame	William Henry Giles Kingston	1870	Non-Canonical
200	Fred Markham in Russia; Or, The Boy Travellers in the Land of the Czar	William Henry Giles Kingston	1858	Non-Canonical
201	In New Granada; Or, Heroes and Patriots	William Henry Giles Kingston	1879	Non-Canonical
202	Roger Kyffin's Ward	William Henry Giles Kingston	1874	Non-Canonical
203	Caxton's Book: A Collection of Essays, Poems, Tales, and Sketches.	William Henry Rhodes	1876	Non-Canonical
204	Blue-Stocking Hall, (Vol. 2 of 3)	William Pitt Scargill	1827	Non-Canonical
205	Blue-Stocking Hall, (Vol. 3 of 3)	William Pitt Scargill	1827	Non-Canonical
206	Aurelian; or, Rome in the Third Century	William Ware	1838	Non-Canonical
207	Scottish Cathedrals and Abbeys	Dugald Butler	1901	Non-Fictional
208	A Text-Book of the History of Architecture: Seventh Edition, revised	Alfred Dwight Foster Hamlin	1896	Non-Fictional
209	Japanese Homes and Their Surroundings	Edward Sylvester Morse	1885	Non-Fictional
210	The Architecture of Provence and the Riviera	David MacGibbon	1888	Non-Fictional
211	Historic Ornament, Vol. 2 (of 2): Treatise on decorative art and architectural ornament	James Ward	1897	Non-Fictional
212	How to Study Architecture	Charles Henry Caffin	1917	Non-Fictional
213	A Dictionary of Slang, Cant, and Vulgar Words: Used at the Present Day in the Streets of London; the Universities of Oxford and Cambridge; the Houses of Parliament; the Dens of St. Giles; and the Palaces of St. James.	John Camden Hotten	1860	Non-Fictional
214	THE ENCYCLOPAEDIA BRITANNICA-Vol 1	University of Cambridge	1910	Non-Fictional
215	THE ENCYCLOPAEDIA BRITANNICA-Vol 2	University of Cambridge	1910	Non-Fictional
216	Through the Brazilian Wilderness	Theodore Roosevelt	1914	Non-Fictional

Continued on next page

Table S1 – *Continued from previous page*

	Title	Author(s)	Year of Publication	Category
217	Gold, Sport, and Coffee Planting in Mysore: With chapters on coffee planting in Coorg, the Mysore representative assembly, the Indian congress, caste and the Indian silver question, being the 38 years' experiences of a Mysore planter	Robert Henry Elliot	1898	Non-Fictional
218	The Economic Aspect of Geology	Charles Kenneth Leith	1921	Non-Fictional
219	The Shores of the Adriatic: The Austrian Side, The Küstenlande, Istria, and Dalmatia	Frederick Hamilton Jackson	1906	Non-Fictional
220	Island Life; Or, The Phenomena and Causes of Insular Faunas and Floras	Alfred Russel Wallace	1880	Non-Fictional
221	Sea and Sardinia	David Herbert Lawrence	1921	Non-Fictional
222	Sketches from the Subject and Neighbour Lands of Venice	Edward Augustus Freeman	1881	Non-Fictional
223	The Principles of Stratigraphical Geology	John Edward Marr	1898	Non-Fictional
224	Babylonian and Assyrian Laws, Contracts and Letters	Claude Hermann Walter Johns	1904	Non-Fictional
225	Putnam's Handy Law Book for the Layman	Albert Sidney Bolles	1921	Non-Fictional
226	Marriage and Divorce Laws of the World	Hyacinthe Ringrose	1911	Non-Fictional
227	The Law and the Poor	Sir Edward Abbott Parry	1914	Non-Fictional
228	International Law	George Grafton Wilson, George Fox Tucker	1901	Non-Fictional
229	The Criminal Prosecution and Capital Punishment of Animals	Edward Payson Evans	1906	Non-Fictional
230	The English Constitution	Walter Bagehot	1867	Non-Fictional
231	The Law of the Sea: A manual of the principles of admiralty law for students, mariners, and ship operators	George L. Canfield, George W. Dalzell, J. Y. Brinton	1921	Non-Fictional
232	Woman and the Republic: A Survey of the Woman-Suffrage Movement in the United States and a Discussion of the Claims and Arguments of Its Foremost Advocates	Helen Kendrick Johnson	1897	Non-Fictional
233	The American Judiciary	Simeon Eben Baldwin	1905	Non-Fictional
234	A Practical Physiology: A Text-Book for Higher Schools	Albert Franklin Blaisdell	1897	Non-Fictional
235	Amusements in Mathematics	Henry Ernest Dudeney	1917	Non-Fictional
236	On the Genesis of Species	St. George Jackson Mivart	1871	Non-Fictional
237	Great Astronomers	Robert Stawell Ball	1895	Non-Fictional
238	Evolution, Old & New: Or, the Theories of Buffon, Dr. Erasmus Darwin and Lamarck; as compared with that of Charles Darwin	Samuel Butler	1879	Non-Fictional
239	Darwin, and After Darwin, Volumes 1 and 3: An Exposition of the Darwinian Theory and a Discussion of Post-Darwinian Questions	George John Romanes	1892	Non-Fictional
240	Creative Evolution	Henri Bergson	1907	Non-Fictional
241	Myths and Marvels of Astronomy	Richard Anthony Proctor	1877	Non-Fictional
242	A Popular History of Astronomy During the Nineteenth Century: Fourth Edition	Agnes Mary AClerke	1887	Non-Fictional
243	A Text-Book of Astronomy	George Cary Comstock	1901	Non-Fictional
244	Astronomical Myths: Based on Flammarions's H́istory of the Heavens´	Camille Flammarion, John Frederick Blake	1877	Non-Fictional
245	Darwin, and After Darwin, Volume 2 of 3: Post-Darwinian Questions: Heredity and Utility	George John Romanes	1892	Non-Fictional
246	A Civic Biology, Presented in Problems	George William Hunter	1914	Non-Fictional
247	Physics	Willis E. Tower, Charles M. Turton, Charles H. Smith, Thomas D. Cope	1920	Non-Fictional
248	A Century of Science, and Other Essays	John Fiske	1899	Non-Fictional
249	Side-Lights on Astronomy and Kindred Fields of Popular Science	Simon Newcomb	1906	Non-Fictional
250	Elementary Zoology, Second Edition	Vernon Lyman Kellogg	1901	Non-Fictional
251	Experiments on Animals	Stephen Paget	1888	Non-Fictional
252	The Sea-beach at Ebb-tide: A Guide to the Study of the Seaweeds and the Lower Animal Life Found Between Tide-marks	Augusta Foote Arnold	1901	Non-Fictional
253	The Science and Philosophy of the Organism	Hans Driesch	1908	Non-Fictional

Continued on next page

Table S1 – *Continued from previous page*

	Title	Author(s)	Year of Publication	Category
254	The Organism as a Whole, from a Physicochemical Viewpoint	Jacques Loeb	1916	Non-Fictional
255	A Guide to the Study of Fishes, Volume 1 (of 2)	David Starr Jordan	1905	Non-Fictional
256	Evolution: Its nature, its evidence, and its relation to religious thought	Joseph LeConte	1888	Non-Fictional
257	The Races of Man: An Outline of Anthropology and Ethnography	Joseph Deniker	1900	Non-Fictional
258	Observations of a Naturalist in the Pacific Between 1896 and 1899, Volume 1: Vanua Levu, Fiji	Henry Brougham Guppy	1903	Non-Fictional
259	Animal Life and Intelligence	Conwy Lloyd Morgan	1890	Non-Fictional
260	Stargazing: Past and Present	Sir Joseph Norman Lockyer	1878	Non-Fictional
261	Observations of a Naturalist in the Pacific Between 1896 and 1899, Volume 2: Plant-Dispersal	Henry Brougham Guppy	1903	Non-Fictional
262	The Logic of Chance, 3rd edition: An Essay on the Foundations and Province of the Theory of Probability, With Especial Reference to Its Logical Bearings and Its Application to Moral and Social Science and to Statistics	John Venn	1888	Non-Fictional
263	Biology and Its Makers: With Portraits and Other Illustrations	William Albert Locy	1908	Non-Fictional
264	The Crayfish: An Introduction to the Study of Zoology.	Thomas Henry Huxley	1880	Non-Fictional
265	History of Botany (1530-1860)	Julius Sachs	1875	Non-Fictional
266	The Universal Kinship	John Howard Moore	1906	Non-Fictional
267	The philosophy of biology	James Johnstone	1914	Non-Fictional
268	Hygienic Physiology : with Special Reference to the Use of Alcoholic Drinks and Narcotics	Joel Dorman Steele	1884	Non-Fictional
269	Species and Varieties, Their Origin by Mutation	Hugo de Vries	1905	Non-Fictional
270	The Naturalist in La Plata	William Henry Hudson	1892	Non-Fictional
271	Studies in the Psychology of Sex, Volume 1: The Evolution of Modesty; The Phenomena of Sexual Periodicity; Auto-Erotism	Havelock Ellis	1900	Non-Fictional
272	Studies in the Psychology of Sex, Volume 2: Sexual Inversion	Havelock Ellis	1900	Non-Fictional
273	The Mind of the Child, Part II: The Development of the Intellect, International Education; Series Edited By William T. Harris, Volume IX.	William T. Preyer	1888	Non-Fictional
274	The Measurement of Intelligence: An Explanation of and a Complete Guide for the Use of the; Stanford Revision and Extension of the Binet-Simon; Intelligence Scale	Lewis Madison Terman	1916	Non-Fictional
275	Human Traits and their Social Significance	Irwin Edman	1919	Non-Fictional
276	Human Personality and Its Survival of Bodily Death	Frederic William Henry Myers	1903	Non-Fictional
277	Mysterious Psychic Forces: An Account of the Author's Investigations in Psychical Research, Together with Those of Other European Savants	Camille Flammarion	1907	Non-Fictional
278	The Group Mind: A Sketch of the Principles of Collective Psychology: With Some Attempt to Apply Them to the Interpretation of National Life and Character	William McDougall	1920	Non-Fictional
279	On the State of Lunacy and the Legal Provision for the Insane: With Observations on the Construction and Organization of Asylums	John Thomas Arlidge	1859	Non-Fictional
280	The Criminal	Havelock Ellis	1890	Non-Fictional
281	Fact and Fable in Psychology	Joseph Jastrow	1900	Non-Fictional
282	A Beginner's Psychology	Edward Bradford Titchener	1915	Non-Fictional
283	The Law of Psychic Phenomena: A working hypothesis for the systematic study of hypnotism, spiritism, mental therapeutics, etc.	Thomson Jay Hudson	1893	Non-Fictional
284	Psychology: Briefer Course	William James	1892	Non-Fictional
285	The Principles of Psychology, Volume 1 (of 2)	William James	1890	Non-Fictional
286	The Principles of Psychology, Volume 2 (of 2)	William James	1890	Non-Fictional
287	Browning as a Philosophical and Religious Teacher	Sir Jones, Henry	1891	Non-Fictional
288	The Life of Reason: The Phases of Human Progress	George Santayana	1905	Non-Fictional
289	An Introduction to Philosophy	George Stuart Fullerton	1906	Non-Fictional
290	The Approach to Philosophy	Ralph Barton Perry	1905	Non-Fictional

Continued on next page

Table S1 – *Continued from previous page*

	Title	Author(s)	Year of Publication	Category
291	The Will to Believe, and Other Essays in Popular Philosophy	William James	1896	Non-Fictional
292	Christianity and Greek Philosophy: or, the relation between spontaneous and reflective thought in Greece and the positive teaching of Christ and His Apostles	Benjamin Franklin Cocker	1870	Non-Fictional
293	A History of Mediaeval Jewish Philosophy	Isaac Husik	1916	Non-Fictional
294	The Philosophy of Friedrich Nietzsche	Henry Louis Mencken	1908	Non-Fictional
295	Philosophical Studies	George Edward Moore	1883	Non-Fictional
296	What Nietzsche Taught	Willard Huntington Wright	1915	Non-Fictional
297	An ethical philosophy of life presented in its main outlines	Felix Adler	1918	Non-Fictional
298	A Beginner's History of Philosophy, Vol. 1: Ancient and Medieval Philosophy	Herbert Ernest Cushman	1910	Non-Fictional
299	Towards the Great Peace	Ralph Adams Cram	1922	Non-Fictional
300	Criminal Man, According to the Classification of Cesare Lombroso	Gina Lombroso	1880	Non-Fictional
301	Criminal Sociology	Enrico Ferri	1895	Non-Fictional
302	Community Civics and Rural Life	Arthur William Dunn	1920	Non-Fictional
303	Sociology and Modern Social Problems	Charles Abram Ellwood	1910	Non-Fictional
304	The Theory of the Leisure Class	Thorstein Veblen	1899	Non-Fictional
305	An Historical View of the Philippine Islands, Vol 1 (of 2): Exhibiting their discovery, population, language, government, manners, customs, productions and commerce.	Joaquin Martinez De Zugniga	1814	Non-Fictional
306	An Historical View of the Philippine Islands, Vol 2 (of 2): Exhibiting their discovery, population, language, government, manners, customs, productions and commerce.	Joaquin Martinez De Zugniga	1814	Non-Fictional
307	History of the Buccaneers of America	James Burney	1816	Non-Fictional
308	The Natural History of Cage Birds: Their Management, Habits, Food, Diseases, Treatment, Breeding, and the Methods of Catching Them.	Johann Matthäus Bechstein	1838	Non-Fictional
309	A System of Pyrotechny: Comprehending the theory and practice, with the application of chemistry; designed for exhibition and for war.	James Cutbush	1825	Non-Fictional
310	The History of the Inquisition of Spain from the Time of its Establishment to the Reign of Ferdinand VII.	Juan Antonio Llorente	1825	Non-Fictional
311	History, Manners, and Customs of the Indian Nations Who Once Inhabited Pennsylvania and the Neighbouring States.	John Gottlieb Ernestus Heckewelder	1818	Non-Fictional
312	On The Principles of Political Economy, and Taxation	David Ricardo	1819	Non-Fictional
313	Pedestrianism; or, An Account of the Performances of Celebrated Pedestrians During the Last and Present Century.: With a full narrative of Captain Barclay's public and private matches; and an essay on training.	Walter Thom	1813	Non-Fictional
314	The Grounds of Christianity Examined by Comparing The New Testament with the Old	George Bethune English	1813	Non-Fictional
315	An Account of The Kingdom of Nepal: And of the Territories Annexed to this Dominion by the House of Gorkha	Francis Hamilton	1819	Non-Fictional
316	The Logic of Hegel	Georg Wilhelm Friedrich Hegel	1812	Non-Fictional
317	Hegel's Philosophy of Mind	Georg Wilhelm Friedrich Hegel	1817	Non-Fictional
318	A Historical Survey of the Customs, Habits, & Present State of the Gypsies	John Hoyland	1816	Non-Fictional
319	Not Paul, But Jesus	Jeremy Bentham	1823	Non-Fictional
320	Aids to Reflection; and, The Confessions of an Inquiring Spirit	Samuel Taylor Coleridge	1825	Non-Fictional
321	The Dance of Death: Exhibited in Elegant Engravings on Wood with a Dissertation on the Several Representations of that Subject but More Particularly on Those Ascribed to Macaber and Hans Holbein	Francis Douce	1833	Non-Fictional

Continued on next page

Table S1 – *Continued from previous page*

	Title	Author(s)	Year of Publication	Category
322	Definitions in Political Economy,: Preceded by an Inquiry Into the Rules which Ought to Guide Political Economists in the Definition and Use of Their Terms; with Remarks on the Deviation from These Rules in Their Writings	Thomas Robert Malthus	1853	Non-Fictional
323	Cottage Economy, to Which is Added The Poor Man's Friend	William Cobbett	1833	Non-Fictional
324	Indian Nullification of the Unconstitutional Laws of Massachusetts Relative to the Marshpee Tribe: Or, the Pretended Riot Explained	William Apess	1835	Non-Fictional
325	Slavery	William Ellery Channing	1835	Non-Fictional
326	Thoughts on Missions	Sheldon Dibble	1850	Non-Fictional
327	A Portraiture of Quakerism, Volume 2: Taken from a View of the Education and Discipline, Social Manners, Civil and Political Economy, Religious Principles and Character, of the Society of Friends	Thomas Clarkson	1841	Non-Fictional
328	The Field Book: or, Sports and pastimes of the United Kingdom: compiled from the best authorities, ancient and modern	William Hamilton Maxwell	1833	Non-Fictional
329	Cosmos: A Sketch of a Physical Description of the Universe	Frédéric Bastiat	1853	Non-Fictional
330	Elements of Physiophilosophy	Lorenz Oken	1847	Non-Fictional
331	A Synopsis of the Birds of North America	Alexander von Humboldt	1845	Non-Fictional
332	The Practical Astronomer: Comprising illustrations of light and colours–practical descriptions of all kinds of telescopes–the use of the equatorial-transit–circular, and other astronomical instruments, a particular account of the Earl of Rosse's large telescopes, and other topics connected with astronomy	Thomas Dick	1850	Non-Fictional
333	The Gastronomic Regenerator: A Simplified and Entirely New System of Cookery: With Nearly Two Thousand Practical Receipts Suited to the Income of All Classes	Alexis Soyer	1846	Non-Fictional
334	The Pantropheon; Or, History of Food, Its Preparation, from the Earliest Ages of the World	Alexis Soyer	1850	Non-Fictional
335	Miss Leslie's New Cookery Book	Eliza Leslie	1867	Non-Fictional
336	Conversations on Chemistry, V. 1-2: In Which the Elements of that Science Are Familiarly Explained and Illustrated by Experiments	Jane Haldimand Marcet	1847	Non-Fictional
337	Conversations on Natural Philosophy, in which the Elements of that Science are Familiarly Explained	Jane Haldimand Marcet	1836	Non-Fictional
338	Botany for Ladies: or, A Popular Introduction to the Natural System of Plants, According to the Classification of De Candolle.	Jane Loudon	1815	Non-Fictional
339	American Institutions and Their Influence	Alexis de Tocqueville	1851	Non-Fictional
340	The Steam Engine Explained and Illustrated (Seventh Edition): With an Account of Its Invention and Progressive Improvement, and Its Application to Navigation and Railways; Including Also a Memoir of Watt	Dionysius Lardner	1840	Non-Fictional
341	History of the State of California: From the Period of the Conquest by Spain to Her Occupation by the United States of America	John Frost	1851	Non-Fictional
342	History of the Conquest of Mexico; vol. 3/4	William Hickling Prescott	1857	Non-Fictional
343	Norman's New Orleans and Environs: Containing a Brief Historical Sketch of the Territory and State of Louisiana and the City of New Orleans, from the Earliest Period to the Present Time	Benjamin Moore Norman	1842	Non-Fictional
344	The Philosophy of Health; Volume 1 (of 2): or, an exposition of the physical and mental constitution of man	Southwood Smith	1847	Non-Fictional
345	History of Brighthelmston; or, Brighton as I View it and Others Knew It: With a Chronological Table of Local Events	John Ackerson Erredge	1851	Non-Fictional

Continued on next page

Table S1 – *Continued from previous page*

	Title	Author(s)	Year of Publication	Category
346	Summary Narrative of an Exploratory Expedition to the Sources of the Mississippi River, in 1820: Resumed and Completed, by the Discovery of its Origin in Itasca Lake, in 1832	Henry Rowe Schoolcraft	1836	Non-Fictional
347	The Infant System: For Developing the Intellectual and Moral Powers of all Children, from One to Seven years of Age	Samuel Wilderspin	1850	Non-Fictional
348	Bulfinch's Mythology: The Age of Fable; The Age of Chivalry; Legends of Charlemagne	Thomas Bulfinch	1862	Non-Fictional
349	Dealings with the Dead, Volume 2 (of 2)	Lucius Manlius Sargent	1846	Non-Fictional
350	Science for the School and Family, Part I. Natural Philosophy	Worthington Hooker	1853	Non-Fictional
351	On the various forces of nature and their relations to each other	Michael Faraday	1847	Non-Fictional
352	British Bees: An Introduction into the Studies of the Natural History and Economy of the Bees Indigenous to the British Isles	William Edward Shuckard	1859	Non-Fictional
353	Gunnery in 1858: Being a Treatise on Rifles, Cannon, and Sporting Arms: Explaining the Principles of the Science of Gunnery, and Describing the Newest Improvements in Fire-Arms	William Greener	1846	Non-Fictional
354	The Sabbath-School Index: Pointing out the history and progress of Sunday-schools, with approved modes of instruction.	Richard Gay Pardee	1842	Non-Fictional
355	The Opium Habit	Horace B. Day	1860	Non-Fictional
356	History of Greece, Volume 12 (of 12)	George Grote	1844	Non-Fictional
357	History of Greece, Volume 03 (of 12)	George Grote	1852	Non-Fictional
358	History of Greece, Volume 05 (of 12)	George Grote	1852	Non-Fictional
359	History of Greece, Volume 04 (of 12)	George Grote	1838	Non-Fictional
360	Reflections on the Decline of Science in England, and on Some of Its Causes	Charles Babbage	1857	Non-Fictional
361	On the Connexion of the Physical Sciences	Mary Somerville	1880	Non-Fictional
362	Knowledge Is Power:: A View of the Productive Forces of Modern Society and the Results of Labor, Capital and Skill.	Charles Knight	1825	Non-Fictional
363	Athens: Its Rise and Fall, Book II	Edward George Bulwer-Lytton	1870	Non-Fictional
364	A Popular History of England, From the Earliest Times to the Reign of Queen Victoria; Vol. I	François Guizot	1837	Non-Fictional
365	Elements of Agricultural Chemistry	Thomas Anderson	1852	Non-Fictional
366	Practical Guide to English Versification: With a Compendious Dictionary of Rhymes, an Examination; of Classical Measures, and Comments Upon Burlesque and; Comic Verse, Vers de Société, and Song-writing	Tom Hood	1838	Non-Fictional
367	A Manual of Elementary Geology: or, The Ancient Changes of the Earth and its Inhabitants as Illustrated by Geological Monuments	Sir Charles Lyell	1844	Non-Fictional
368	Health and Education	Charles Kingsley	1837	Non-Fictional
369	Stones of the Temple; Or, Lessons from the Fabric and Furniture of the Church	Walter Field	1847	Non-Fictional
370	History of the United Netherlands from the Death of William the Silent to the Twelve Year's Truce — Complete (1600-1609)	John Lothrop Motley	1849	Non-Fictional
371	A Dictionary of English Synonymes and Synonymous or Parallel Expressions: Designed as a Practical Guide to Aptness and Variety of Phraseology	Richard Soule	1838	Non-Fictional
372	The Rise of the Dutch Republic — Complete (1566-74)	John Lothrop Motley	1871	Non-Fictional
373	A History of Domestic Manners and Sentiments in England During the Middle Ages	Thomas Wright	1859	Non-Fictional
374	The History, Theory, and Practice of Illuminating: Condensed from 'The Art of Illuminating' by the same illustrator and author	Matthew Digby Wyatt	1860	Non-Fictional
375	An Architect's Note-Book in Spain: principally illustrating the domestic architecture of that country.	Matthew Digby Wyatt	1858	Non-Fictional
376	History of Lace	Fanny Bury Palliser	1862	Non-Fictional

Continued on next page

Table S1 – *Continued from previous page*

	**Title**	**Author(s)**	**Year of Publication**	**Category**
377	The Physical Basis of Mind: Being the Second Series of Problems of Life and Mind.	George Henry Lewes	1853	Non-Fictional
378	Lectures on the rise and development of medieval architecture; vol. 2	George Gilbert Scott	1872	Non-Fictional
379	The History of Ancient America, Anterior to the Time of Columbus: Proving the Identity of the Aborigines with the Tyrians and Israelites; and the Introduction of Christianity into the Western Hemisphere By The Apostle St. Thomas	George Jones	1838	Non-Fictional
380	The Subterranean World	Georg Hartwig	1871	Non-Fictional
381	Guano: A Treatise of Practical Information for Farmers	Solon Robinson	1852	Non-Fictional
382	Wild Wales: The People, Language, & Scenery	George Borrow	1862	Non-Fictional
383	History of Indian and Eastern Architecture	James Fergusson	1876	Non-Fictional
384	Parasites: A Treatise on the Entozoa of Man and Animals: Including Some Account of the Ectozoa	Thomas Spencer Cobbold	1879	Non-Fictional
385	History of American Socialisms	John Humphrey Noyes	1869	Non-Fictional
386	London Labour and the London Poor, Vol. 2	Henry Mayhew	1851	Non-Fictional
387	Companion to the Bible	Elijah Porter Barrows	1867	Non-Fictional
388	The Non-religion of the Future: A Sociological Study	Jean-Marie Guyau	1887	Non-Fictional
389	Ten Great Religions: An Essay in Comparative Theology	James Freeman Clarke	1871	Non-Fictional
390	A History of Oregon, 1792-1849: Drawn From Personal Observation and Authentic Information	William Henry Gray	1870	Non-Fictional
391	Bible Animals;: Being a Description of Every Living Creature Mentioned in the Scripture, from the Ape to the Coral.	John George Wood	1869	Non-Fictional

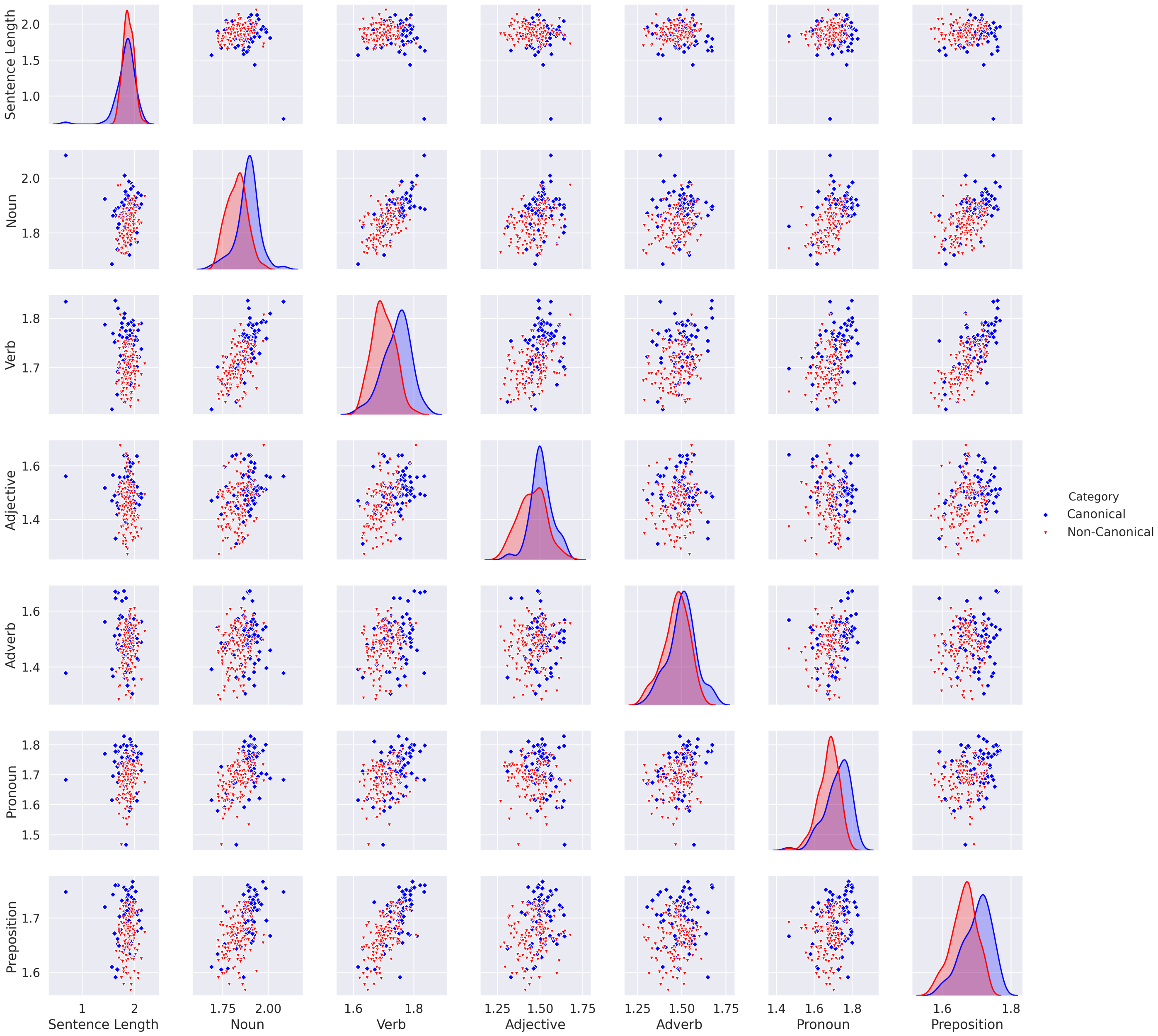

Figure S1. Pair-plot of all Approximate Entropy (ApEn) features in fictional/canonical and fictional/non-canonical texts. While each non-diagonal plot shows the relationships between two features, the main-diagonal subplots visualize the univariate distributions of each feature.

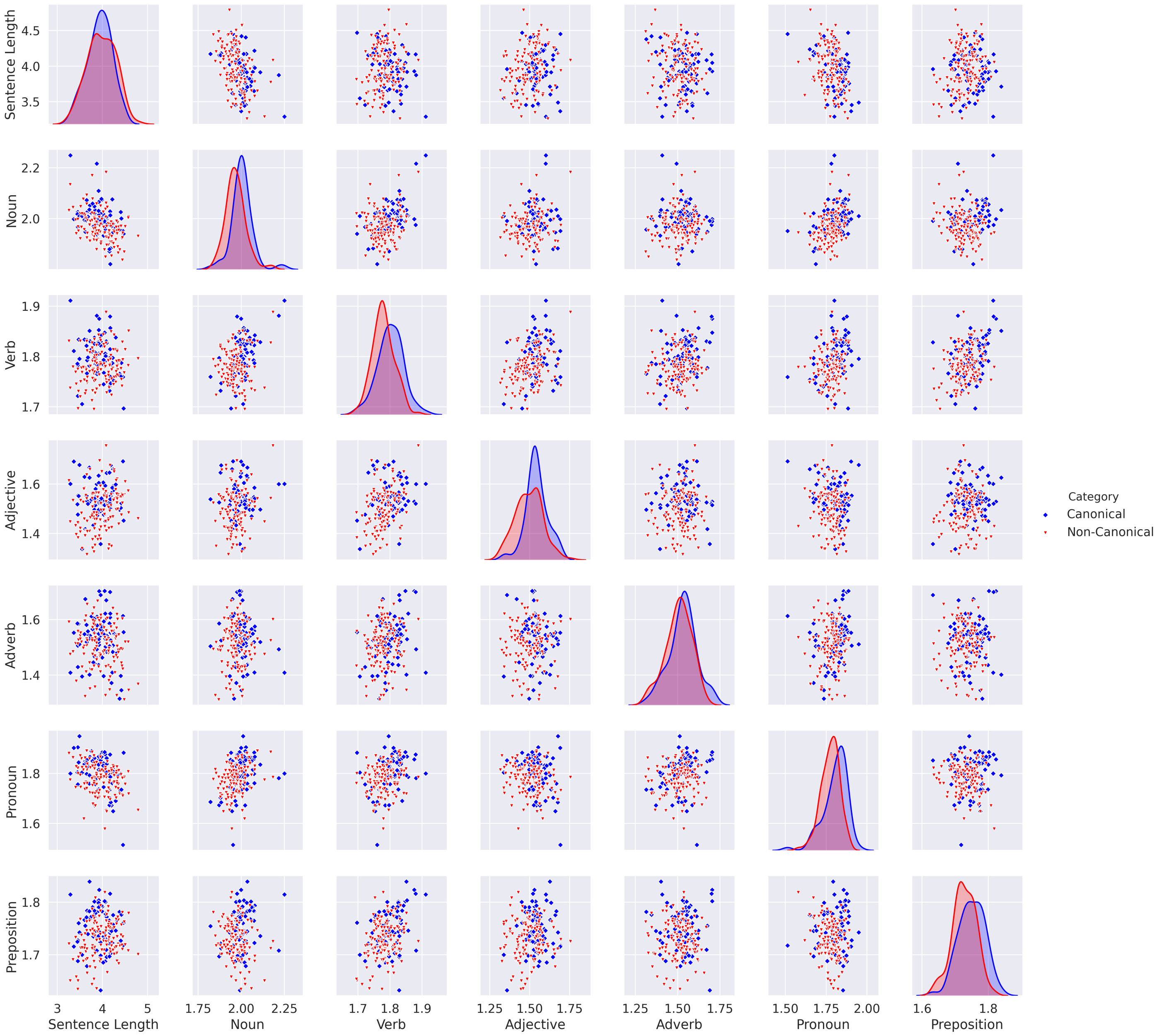

Figure S2. Pair-plot of all Shannon Entropy (ShEn) features in fictional/canonical and fictional/non-canonical texts. While each non-diagonal plot shows the relationships between two features, the main-diagonal subplots visualize the univariate distributions of each feature.

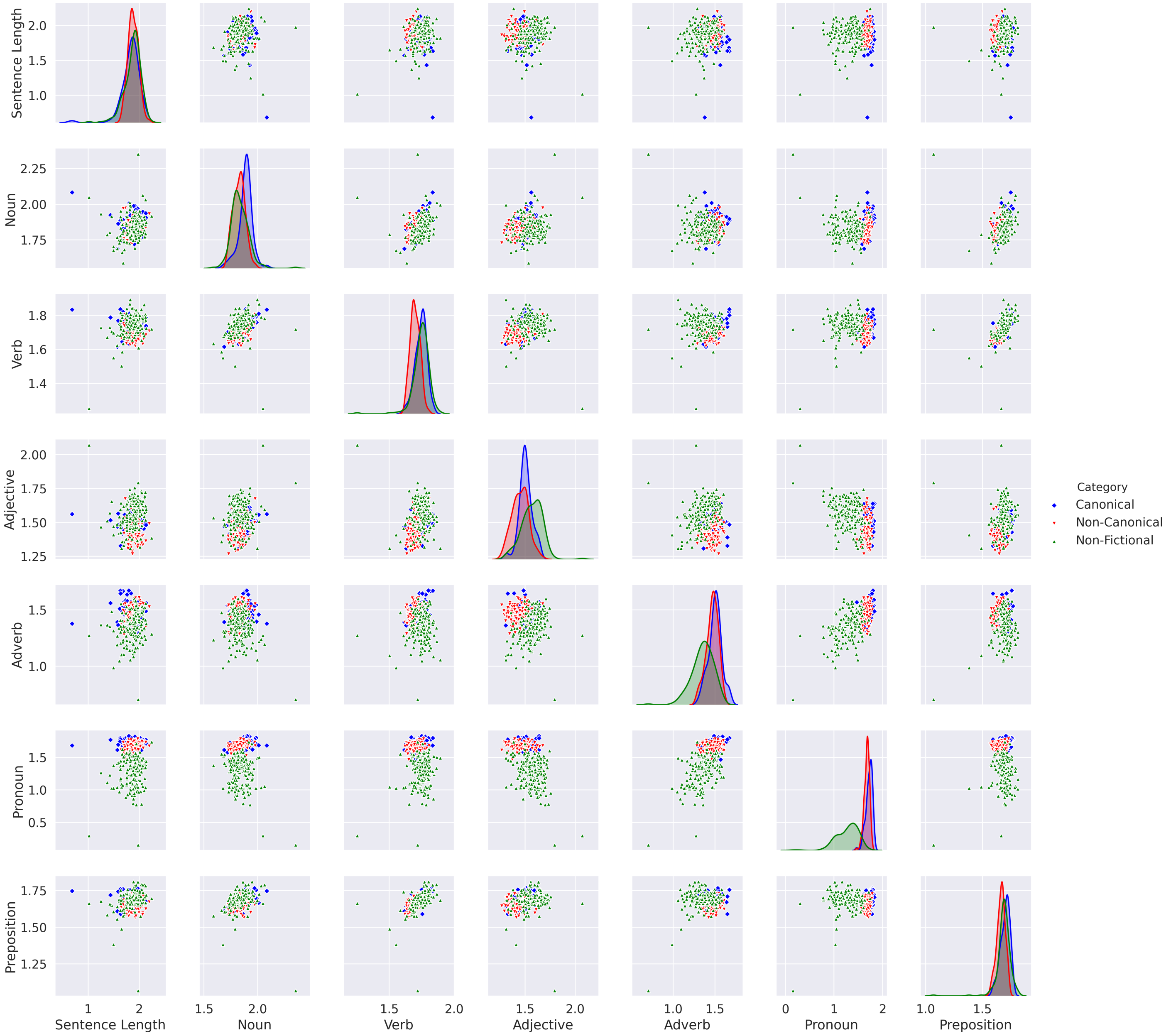

Figure S3. Pair-plot of all Approximate Entropy (ApEn) features in fictional/canonical, fictional/non-canonical and non-fictional texts. For better visibility of the data for canonical texts vs. non-canonical texts, see Figure S1. While each non-diagonal plot shows the relationships between two features, the main-diagonal subplots visualize the univariate distributions of each feature.

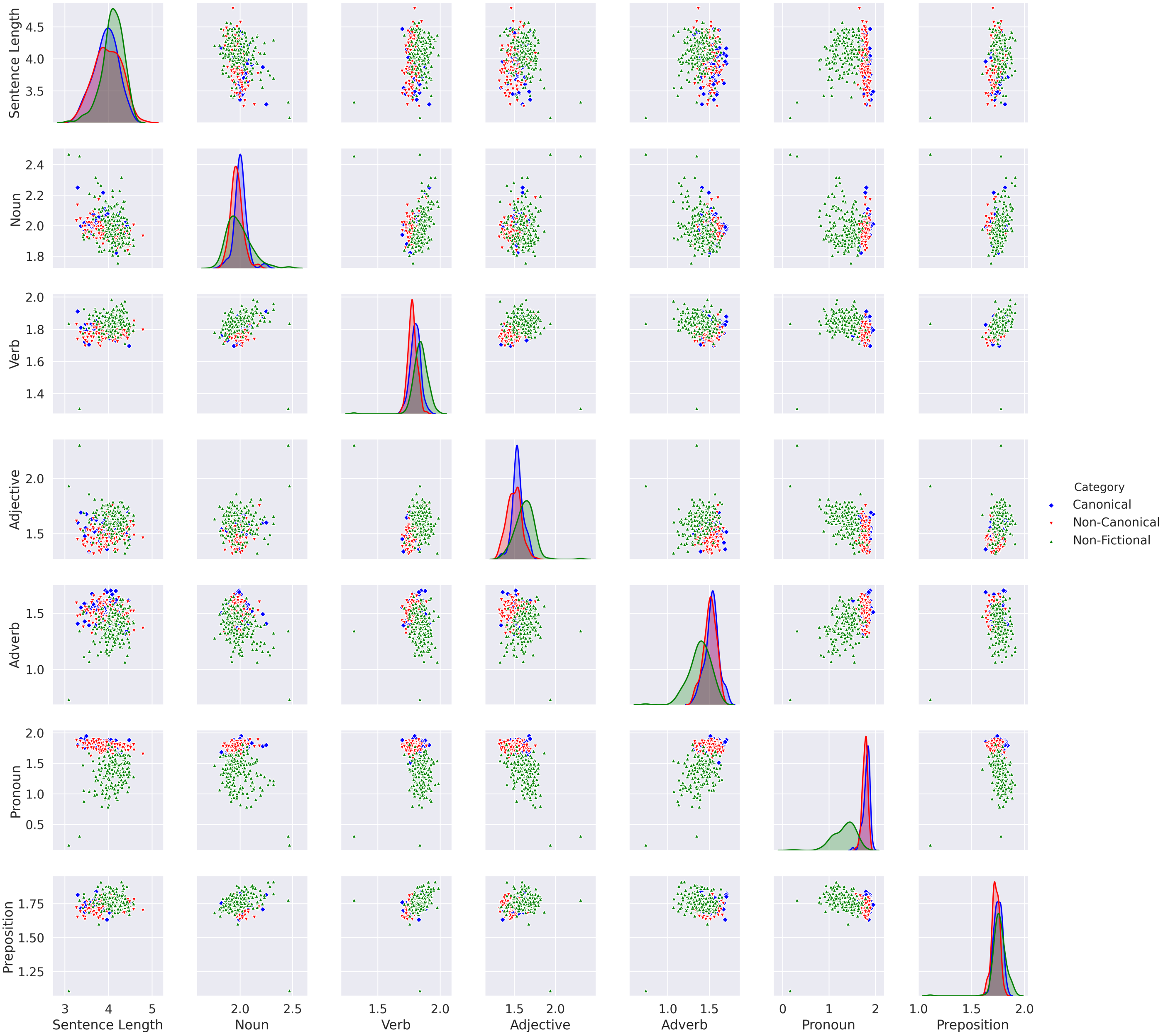

Figure S4. Pair-plot of all Shannon Entropy (ShEn) features in fictional/canonical, fictional/non-canonical and non-fictional texts. For better visibility of the data for canonical texts vs. non-canonical texts, see Figure S2. While each non-diagonal plot shows the relationships between two features, the main-diagonal subplots visualize the univariate distributions of each feature.

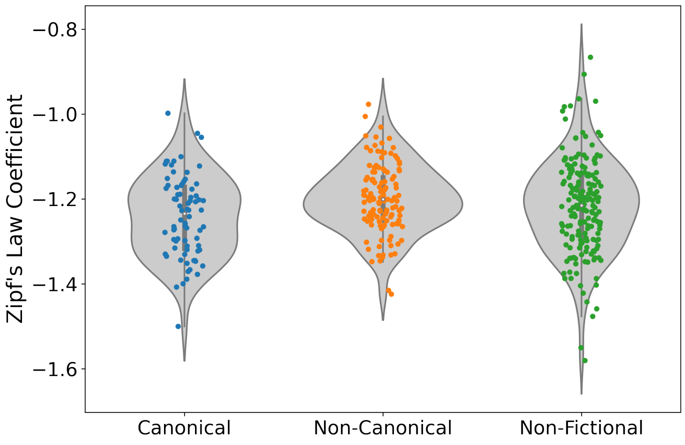

Figure S5. Zipf's law coefficient (lambda) of fictional/canonical, fictional/non-canonical and non-fictional texts. The high overlap of values between the text categories results in a poor classification accuracy.

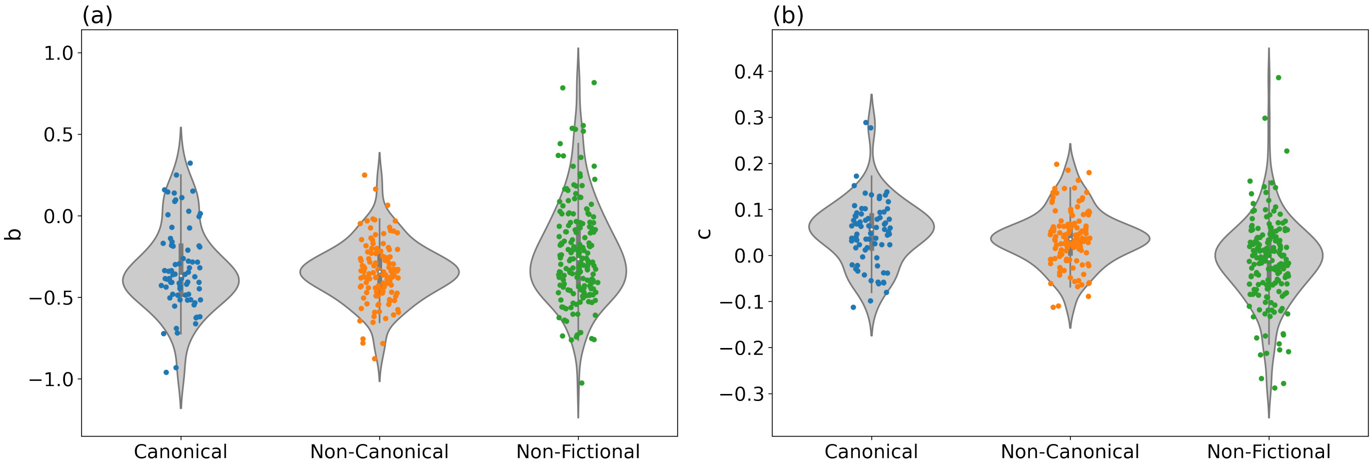

Figure S6. Menzerath–Altmann law assumes a relation between the size of constituents, y, of a linguistic construct with the size of the construct, x: $y = ax^b e^{-cx}$. The plots in (a) and (b) represent the two parameters of the Manzerath-Altmann law, b and c, respectively, in fictional/canonical, fictional/non-canonical and non-fictional texts.

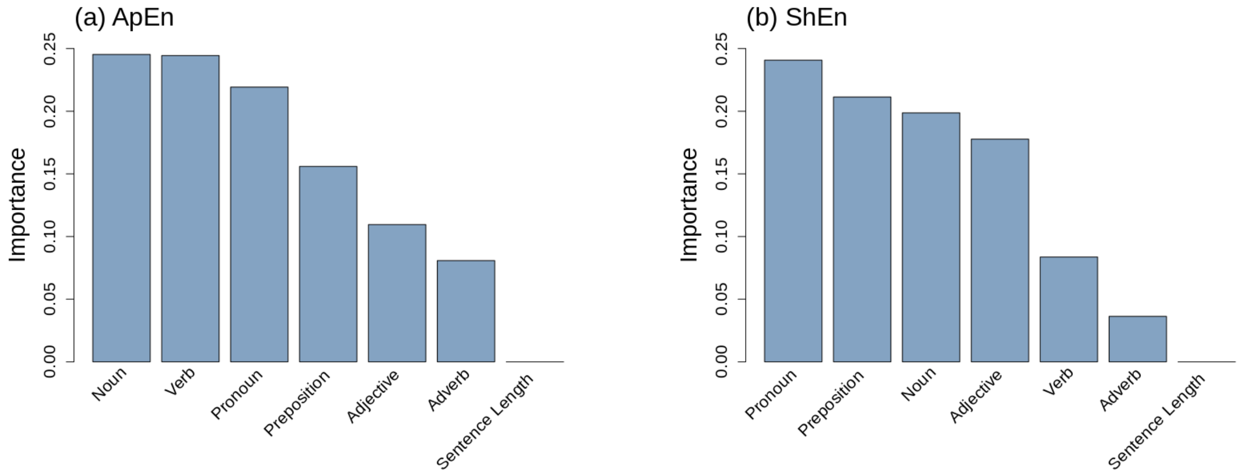

Figure S7. Sensitivity analysis of ApEn features (a) and ShEn features (b) in classification of fictional/canonical and fictional/non-canonical texts.

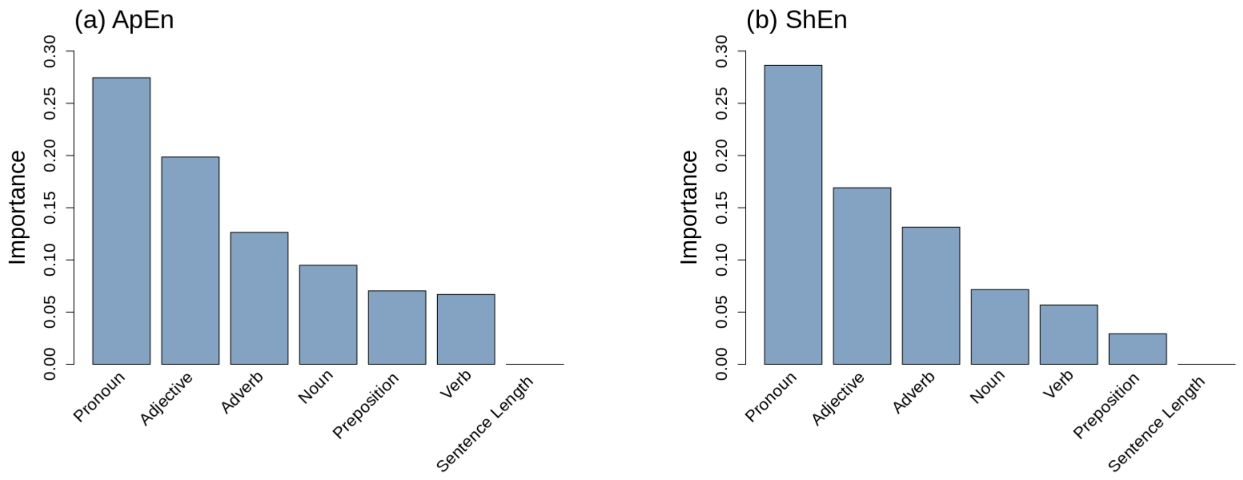

Figure S8. Sensitivity analysis of ApEn features (a) and ShEn features (b) in classification of fictional and non-fictional texts.
